# LEA motifs promote desiccation tolerance in vivo

**DOI:** 10.1186/s12915-021-01176-0

**Published:** 2021-12-14

**Authors:** Jonathan D. Hibshman, Bob Goldstein

**Affiliations:** 1 Biology Department, University of North Carolina at Chapel Hill, Chapel Hill, NC 27599-3280 USA; 2 Lineberger Comprehensive Cancer Center, University of North Carolina at Chapel Hill, Chapel Hill, NC USA

**Keywords:** Late embryogenesis abundant (LEA), Desiccation, Osmotic stress, *C. elegans*, Stress

## Abstract

**Background:**

Cells and organisms typically cannot survive in the absence of water. However, some animals including nematodes, tardigrades, rotifers, and some arthropods are able to survive near-complete desiccation. One class of proteins known to play a role in desiccation tolerance is the late embryogenesis abundant (LEA) proteins. These largely disordered proteins protect plants and animals from desiccation. A multitude of studies have characterized stress-protective capabilities of LEA proteins in vitro and in heterologous systems. However, the extent to which LEA proteins exhibit such functions in vivo, in their native contexts in animals, is unclear. Furthermore, little is known about the distribution of LEA proteins in multicellular organisms or tissue-specific requirements in conferring stress protection. Here, we used the nematode *C. elegans* as a model to study the endogenous function of an LEA protein in an animal.

**Results:**

We created a null mutant of *C. elegans* LEA-1, as well as endogenous fluorescent reporters of the protein. LEA-1 mutant animals formed defective dauer larvae at high temperature. We confirmed that *C. elegans* lacking LEA-1 are sensitive to desiccation. LEA-1 mutants were also sensitive to heat and osmotic stress and were prone to protein aggregation. During desiccation, LEA-1 expression increased and became more widespread throughout the body. LEA-1 was required at high levels in body wall muscle for animals to survive desiccation and osmotic stress, but expression in body wall muscle alone was not sufficient for stress resistance, indicating a likely requirement in multiple tissues. We identified minimal motifs within *C. elegans* LEA-1 that were sufficient to increase desiccation survival of *E. coli*. To test whether such motifs are central to LEA-1’s in vivo functions, we then replaced the sequence of *lea-1* with these minimal motifs and found that *C. elegans* dauer larvae formed normally and survived osmotic stress and mild desiccation at the same levels as worms with the full-length protein.

**Conclusions:**

Our results provide insights into the endogenous functions and expression dynamics of an LEA protein in a multicellular animal. The results show that LEA-1 buffers animals from a broad range of stresses. Our identification of LEA motifs that can function in both bacteria and in a multicellular organism in vivo suggests the possibility of engineering LEA-1-derived peptides for optimized desiccation protection.

**Supplementary Information:**

The online version contains supplementary material available at 10.1186/s12915-021-01176-0.

## Background

Animals regularly encounter abiotic stresses due to fluctuating environments. One such stress is desiccation. Water is essential for cellular metabolism, and without it cellular components including DNA, RNA, proteins, and membranes are unstable. However, certain animals can lose nearly all their internal water and yet survive (anhydrobiosis). This group includes nematodes, rotifers, tardigrades, and certain arthropods such as some crustaceans and insects. Studying the mechanisms by which these organisms are able to survive desiccation is of fundamental interest and may lead to better understanding of biological protectants.

One family of common protectants with demonstrated efficacy during desiccation is the late embryogenesis abundant (LEA) proteins. These proteins were originally identified in cotton seeds and were subsequently found to be present in many other plants [1–6]. LEA proteins protect the viability of desiccated seeds [7]. LEA proteins were later identified in the nematode *Aphelenchus avenae* and other anhydrobiotic animals [8–13]. Several families of LEA proteins have been described, each containing distinct motifs [14, 15]. Nematode LEA proteins both in *A. avenae* as well as the more common model system *C. elegans* are members of the Group 3 LEA proteins. This is the most common LEA variety within anhydrobiotic animals [16]. LEA proteins are thought to be intrinsically disordered, i.e., largely unstructured in hydrated conditions, but under water-limiting conditions the proteins acquire recognizable alpha-helical secondary structure [17–19]. There are several examples in which heterologous expression of LEA proteins confers stress resistance in a number of host cell types, including yeast, bacteria, rice, *D. melanogaster*, and mammalian cell culture [20–26]. Additionally, isolated LEA protein has been demonstrated to protect against protein aggregation and stabilize membranes and liposomes in vitro [27–29]. Although the functions of LEA proteins are well-established, most studies have utilized heterologous expression systems. Therefore, we have limited knowledge of the endogenous, in vivo roles of LEA proteins.

The nematode *C. elegans* is an ideal model for the study of the endogenous functions of LEA proteins. *C. elegans* survive many stresses including desiccation, have a well-resourced genetic toolkit, and contain two LEA family proteins, *lea-1* and *dur-1* (dauer upregulated) [10, 30]. Disruption of *lea-1* has been reported to sensitize worms to desiccation, osmotic stress with sucrose, and larval heat stress [10, 30, 31]. *dur-1* is also required for a robust desiccation response but is apparently unable to compensate for the loss of LEA-1 [30]. Whereas most studies show that heterologous expression is sufficient to protect against stresses in other systems, these *C. elegans* studies provide evidence for necessity in the native function of LEA proteins in animals. However, little is known about the tissues in which LEA proteins are required or the mechanisms by which they function in vivo [14, 32, 33].

We used LEA-1 of *C. elegans* as a model to study the endogenous function of an LEA protein in a multicellular animal. We confirmed and expanded phenotypes associated with disruption of LEA-1, characterized expression patterns and dynamics in response to desiccation, and identified minimal amino acid motifs within the protein that harbor the biochemical properties to confer desiccation resistance. These results provide insights into the in vivo, endogenous functions of an LEA protein in a multicellular animal.

## Results

### Generation of a null allele of *lea-1*

**Fig. 1:**
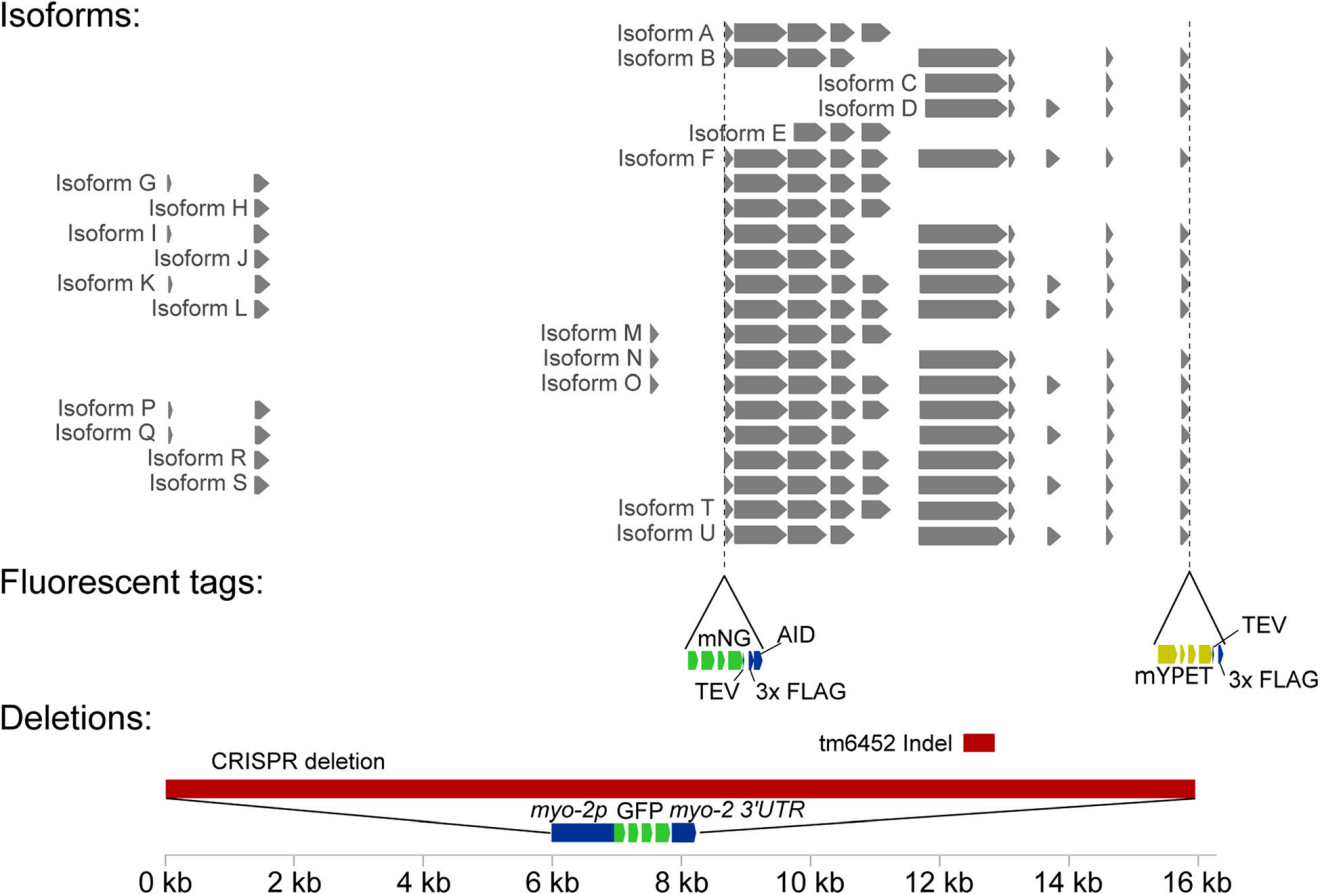
Predicted isoform structure of LEA-1 and generation of a null allele and two endogenous fluorescent tags. Isoform annotations were combined from the UCSC genome browser (http://genome.ucsc.edu) from the February 2013 release (WBcel235/ce11) and Wormbase version WS278. Locations for the existing tm6452 indel mutation, the newly created CRISPR null mutation, and the insertion of mNeonGreen and mYPET fluorescent tags are indicated

We were motivated to determine the role of LEA-1 in the endogenous organismal context of *C. elegans*. Previous studies have relied on RNAi for gene knockdown and did not fully eliminate *lea-1* mRNA [10] or noted that RNAi seldom fully eliminates the target mRNA [30]. One recent study used a deletion allele, but only confirmed the phenotype of desiccation sensitivity [31]. Furthermore, there are many isoforms of LEA-1 (Fig. 1), and the potential for alternative isoform usage makes mutant analysis difficult to interpret. To address these concerns, we created a full-length, 15.8 kb deletion of the *lea-1* locus via CRISPR, and we also cleaned the genetic background of a second, pre-existing mutant allele, *lea-1(tm6452)*, by outcrossing it to wild-type worms for 5 generations [10, 30, 34, 35]. For the full-length deletion (*lea-1Δ*), the deleted gene was replaced with a *pmyo-2*::GFP::*myo-2 3′-UTR* construct as a reporter for the deletion (Fig. 1). The successful deletion was confirmed via visualization of pharyngeal expression of green fluorescent protein (GFP) as well as by PCR (Additional File 1: Fig. S1A-D).

### *lea-1* mutants exhibit a temperature-dependent defect in forming normal dauer larvae

**Fig. 2:**
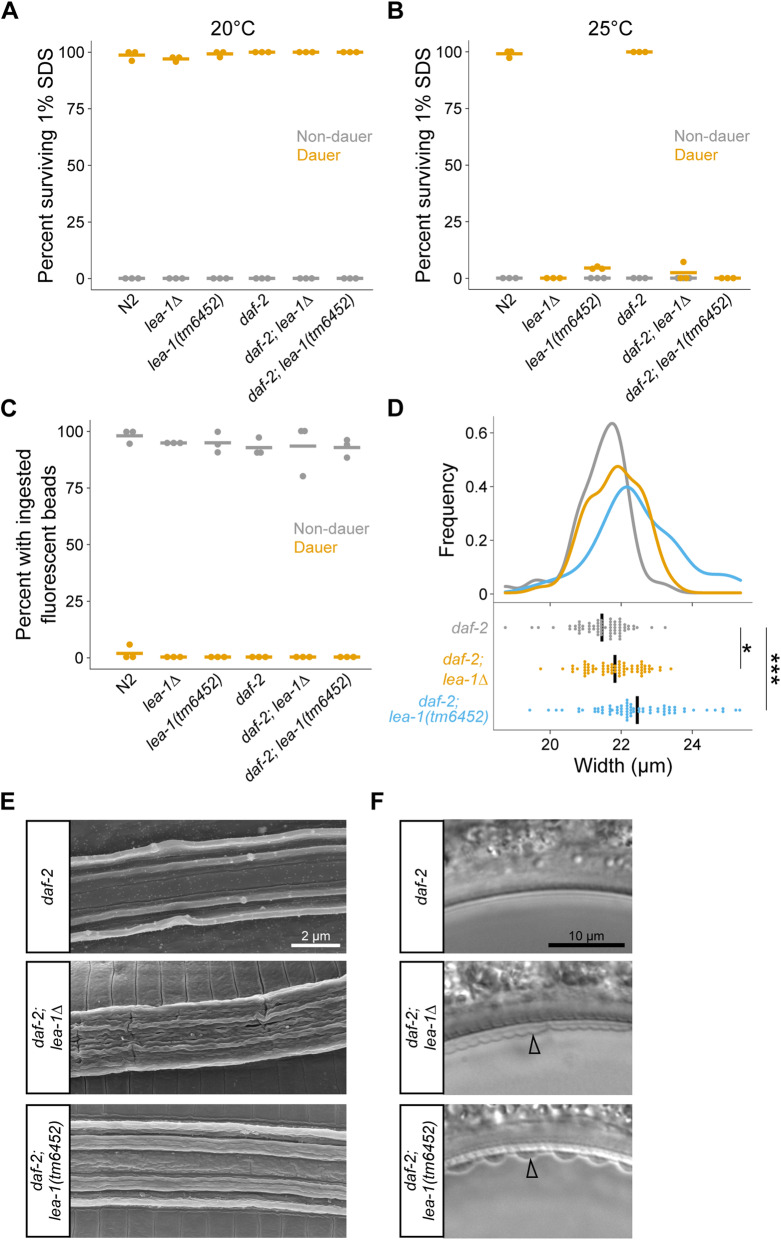
*lea-1* mutants have a temperature-sensitive dauer formation defect. **A** Dauer larvae formed at 20 °C are resistant to 1% SDS. **B** Dauer larvae of *lea-1Δ* and *lea-1(tm6452)* mutants formed at 25 °C are sensitive to 1% SDS in both a wild-type (N2) background and a *daf-2(e1370)* mutant background. **C** *lea-1Δ*, *lea-1(tm6452)*, *daf-2*;*lea-1Δ* and *daf-2;lea-1(tm6452)* mutant dauer-like larvae formed at 25 °C do not ingest fluorescent beads. Non-dauer larvae of each genotype did exhibit feeding behavior and ingest beads. **D** *daf-2*;*lea-1Δ* and *daf-2;lea-1(tm6452)* mutants at 25 °C have limited radial constriction relative to *daf-2* mutant dauers. A one-way ANOVA indicates significant differences across genotypes (*p* < 0.0001) and a post-hoc Dunnett’s test indicates that both *daf-2;lea-1Δ* and *daf-2;lea-1(tm6452)* had increased width relative to *daf-2* controls (*p* = 0.04, *p* < 0.0001, respectively). The width of dauer larvae was measured posterior to the pharynx. Points represent the width of individual worms and black bars depict the mean. The distribution of widths is shown above in density plots. **E** SEM images highlight the ultrastructure of alae of *daf-2*, *daf-2*;*lea-1Δ* and *daf-2;lea-1(tm6452)* mutants. *daf-2* mutant dauers display normal alae consisting of five distinct ridges. Each of the *lea-1* mutants have disrupted alae. **F** DIC images show *daf-2*;*lea-1Δ* and *daf-2;lea-1(tm6452)* dauer-like larvae with detached cuticles, as indicated by arrowheads

LEA proteins are known for promoting survival during desiccation. Because *C. elegans* are reported to survive desiccation only when in the dauer state [36], we wanted to determine if LEA-1 impacts dauer formation. Multiple environmental variables influence dauer development, including population density, food availability, and temperature [37]. Additionally, some genetic mutants constitutively form dauer larvae. For example, *daf-2(e1370)* mutant animals are commonly used to obtain dauer larvae as they develop into dauer larvae when grown at 25 °C [38]. Dauer larvae cease feeding, constrict along the radial axis, form a buccal plug, and synthesize a thickened cuticle with lateral ridges called alae—adaptations that limit exposure to environmental insults [37, 39–41]. For example, dauer larvae are able to survive exposure to the detergent sodium dodecyl sulfate (SDS) [39, 42]. Because it was possible that LEA-1 limits desiccation tolerance by disrupting proper dauer formation, we assessed dauer traits of *lea-1* mutants in both wild-type (N2) and *daf-2(e1370)* backgrounds. When worms were grown at 20 °C, dauer larvae were identifiable as population density increased and food was depleted. *lea-1(tm6452)* and *lea-1Δ* dauer larvae that formed in these conditions were resistant to 1% SDS in both wild-type and *daf-2* backgrounds (Fig. 2A). However, *lea-1(tm6452)* and *lea-1Δ* worms cultured at 25 °C died when exposed to 1% SDS, unlike N2 or *daf-2* control animals cultured at 25 °C (Fig. 2B). Temperature-dependent sensitivity to 1% SDS was independent of the method of dauer formation (crowding and starvation in an N2 background vs. constitutive dauer formation at 25 °C in a *daf-2* background).

To determine the nature of the dauer defect of *lea-1* mutant animals cultured at 25 °C, we measured several dauer-specific traits. *lea-1* mutants exhibited normal arrest behavior without feeding. Like control animals, *lea-1Δ*, *lea-1(tm6452)*, *daf-2;lea-1Δ*, and *daf-2;lea-1(tm6452)* mutants did not exhibit feeding behavior as dauer-like larvae, as measured by ingestion of fluorescent beads (Fig. 2C). In a *daf-2* background, each mutant allele of *lea-1* caused a modest increase in the width of dauer-like larvae, suggesting a slight defect in radial constriction (Fig. 2D). *lea-1* mutant dauer-like larvae appear to arrest similarly to controls, with sealed mouths and lateral alae (Additional File 1: Fig. S2). Because the structure of dauer-specific alae was difficult to assess with DIC optics, we used scanning electron microscopy to assess the ultrastructure of alae. We observed a disruption in the structure of alae, with alae of mutant animals having less-defined ridges (Fig. 2E). Additionally, the cuticle of *lea-1* mutant animals was prone to detachment (Fig. 2F). Cuticle detachment was seen most prominently at bends in the animals. Collectively, these observations define an in vivo function for *lea-1* by showing that dauer larvae form in *lea-1* mutants, but in the larvae that form at 25 °C, cuticle formation and radial constriction are disrupted, and animals are sensitive to 1% SDS.

### *lea-1* deletion mutants are sensitive to multiple stresses

**Fig. 3:**
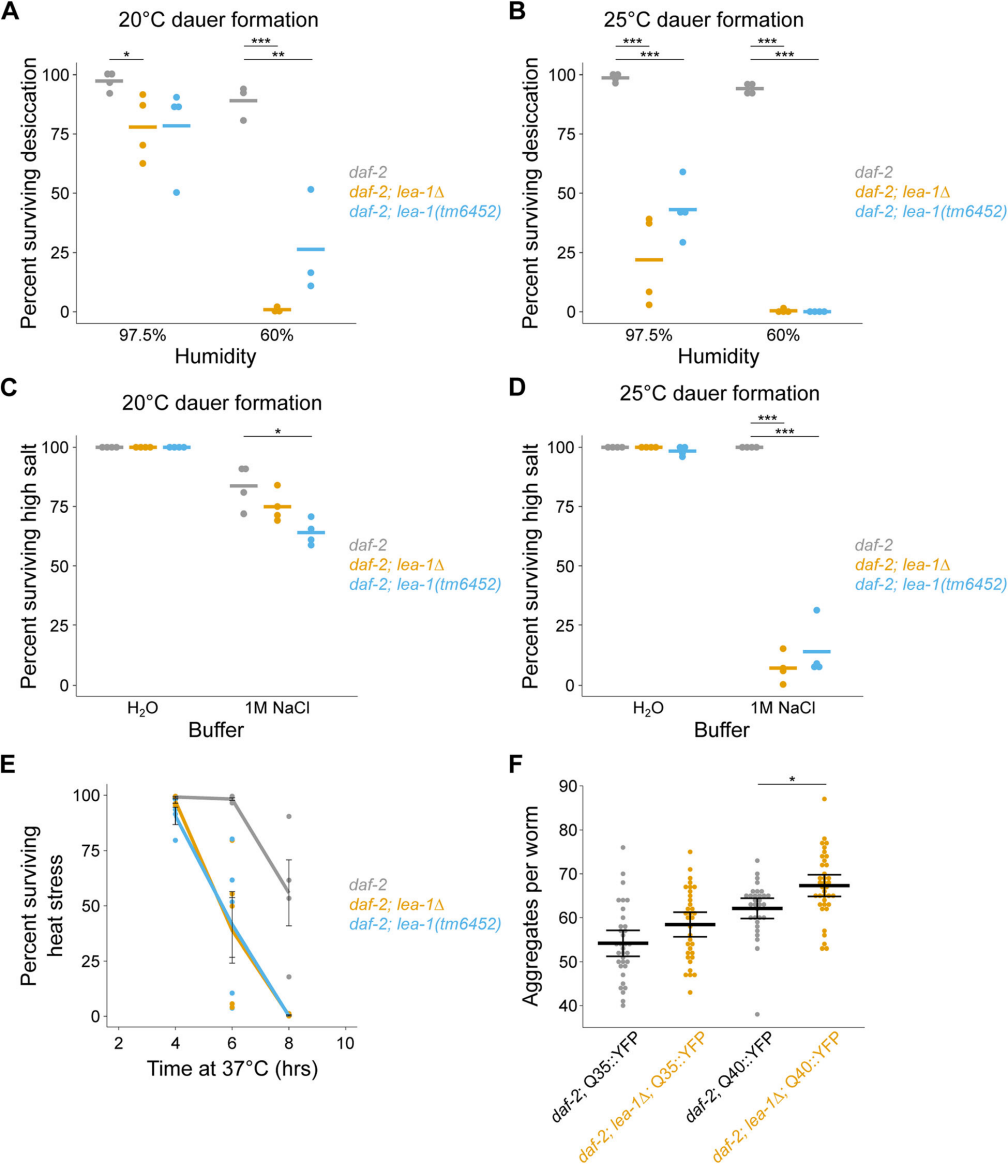
LEA-1 is required for resistance to multiple stresses. **A** *daf-2;lea-1Δ* and *daf-2;lea-1(tm6452)* dauer larvae formed at 20 °C are sensitive to desiccation. Survival is modestly reduced in worms exposed to 97.5% relative humidity for 4 days (*p* = 0.03, *p* = 0.10 respectively, *n* = 4, unpaired *T* test) and more significantly reduced after an additional day at 60% RH (*p* < 0.0001, *p* = 0.009 respectively, *n* = 3, unpaired *T* test). **B** Dauer-like larvae of *daf-2;lea-1Δ* and *daf-2;lea-1(tm6452)* mutants formed at 25 °C are sensitive to desiccation. Survival is significantly reduced in worms exposed to 97.5% relative humidity for 4 days (*p* = 0.0002, *p* = 0.0001 respectively, *n* = 4, unpaired *T* test) as well as 60% for 1 day after 4 days at 97.5% (*p* < 0.0001, *p* < 0.0001, *n* = 4, unpaired *T* test). **C** *lea-1* mutant dauers formed at 20 °C exhibit mild sensitivity to osmotic stress in 1 M NaCl for 2 h (*daf-2;lea-1Δ p* = 0.17, *daf-2;lea-1(tm6452) p* = 0.02, *n* = 4, unpaired *T* test). **D** Dauer-like larvae of *daf-2;lea-1Δ* and *daf-2;lea-1(tm6452)* mutants are sensitive to osmotic stress in 1 M NaCl for 2 h (*p* < 0.0001 for each genotype compared to control, *n* = 4, unpaired *T* test). Data points in A–D represent results from individual experiments and bars represent mean survival. **E** Dauer larvae of *lea-1Δ* and *lea-1(tm6452)* mutants are sensitive to heat stress at 37 °C (*p* < 0.0001 for each mutant allele relative to control, *n* = 4–5 replicates per timepoint, 2-way ANOVA). Lines depict mean survival and error bars represent SEM. Data points depict survival from individual experiments. **F** *daf-2;lea-1Δ* mutants have a higher number of polyglutamine protein aggregates. The total number of aggregates in the body wall muscle of individual worms from three independent biological replicates is shown. Thick bars indicate the mean number of aggregates and error bars depict 95% confidence intervals. Worms carrying a transgene expressing a 35-glutamine repeat (Q35) with a YFP for visualization have only marginally more aggregates than controls (*p* = 0.22, *n* = 3, unpaired *T* test), while worms carrying a Q40::YFP transgene have significantly more aggregates than controls (*p* = 0.02, *n* = 3, unpaired *T* test). *p < 0.05, **p < 0.01, ***p < 0.001

To determine the impact of defective dauer formation on stress resistance, we measured survival of desiccation and osmotic stress in dauer or dauer-like larvae formed at 20 °C or 25 °C. We chose to conduct the majority of our experiments in the temperature-sensitive constitutive dauer *daf-2(e1370)* background to facilitate consistent production of dauer worms. In a *daf-2* background, *lea-1Δ* and *lea-1(tm6452)* mutant dauer larvae formed at 20 °C (SDS-resistant) were only mildly sensitive to desiccation at high relative humidity (97.5%), but were highly sensitive to a more stringent desiccation stress of 60% humidity (Fig. 3A), consistent with published results [10, 30, 31]. When dauer-like larvae formed at 25 °C were exposed to the same conditions, *daf-2;lea-1Δ* and *daf-2;lea-1(tm6452)* mutant animals had lower survival than controls, even at 97.5% RH (Fig. 3B). Similar results were also observed in an N2 genetic background (Additional File 1: Fig. S3A,B). This suggests that defective dauer formation of *lea-1* mutants at 25 °C does not fully explain the phenotype of desiccation sensitivity, but can compound its severity.

Additionally, dauer worms of each of these mutant strains were sensitive to osmotic stress in a concentrated salt solution. While there were no differences in survival in worms kept in water for 2 h, *daf-2;lea-1(tm6452)* mutant dauers formed at 20 °C had slightly lower survival in 1 M NaCl for 2 h (Fig. 3C). Both *daf-2;lea-1Δ* and *daf-2;lea-1(tm6452)* dauer-like larvae formed at 25 °C had significantly lower survival when exposed to 1 M NaCl for 2 h (Fig. 3D). Each *lea-1* mutant strain was also sensitive to 1 M NaCl in a wild-type (non-*daf-2*) genetic background (Additional File 1: Fig. S3C,D). We also found that *daf-2;lea-1Δ* and *daf-2;lea-1(tm6452)* mutant dauer-like larvae formed at 25 °C were sensitive to exposure to heat shock at 37 °C (Fig. 3E).

LEA proteins have been demonstrated to prevent protein aggregation in vitro and in cell culture [27, 43–45]. To test for in vivo roles in preventing protein aggregation, we assessed the number of polyglutamine aggregates in body wall muscle of *daf-2;lea-1Δ* dauer-like animals (25 °C dauer formation). These animals expressed tracts of 35 or 40 glutamines fused to a yellow fluorescent protein (YFP) reporter [46]. Mutants with the Q35::YFP reporter did not have a statistically significant difference from controls (*p* = 0.22); however, *daf-2;lea-1Δ* animals with a Q40::YFP reporter had significantly more aggregates per worm than controls (*p* = 0.02) after 5 days of development and dauer formation (Fig. 3F). We conclude that LEA-1 has in vivo functions protecting animals against desiccation, osmotic stress, heat stress, and formation of polyglutamine protein aggregates and that temperature-sensitive defects in dauer formation at 25 °C can contribute to but do not fully explain the stress sensitivity of *lea-1* mutants.

### LEA-1 lacks discernable nonredundant functions outside of stress tolerance

**Fig. 4:**
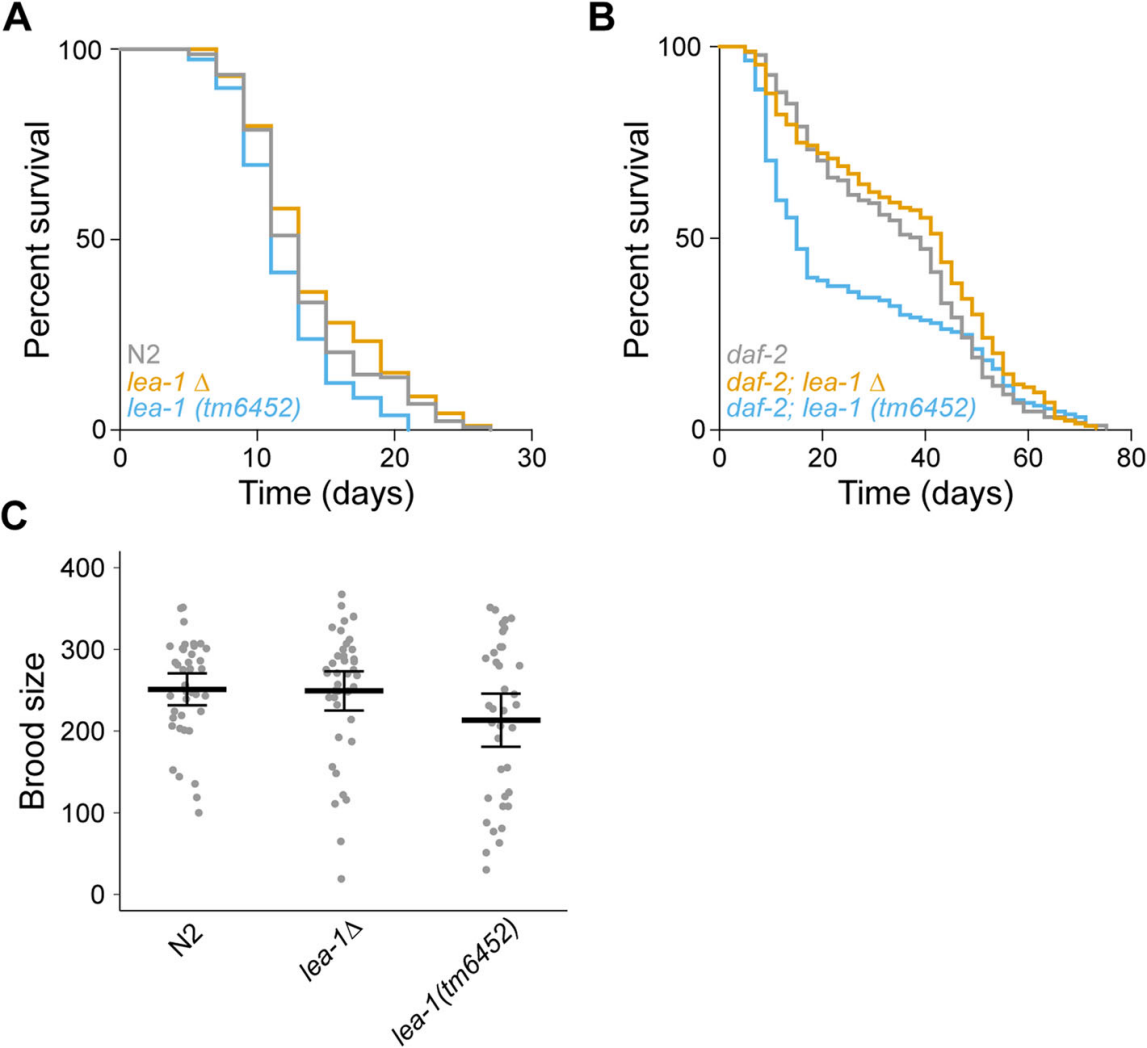
Phenotypes of LEA-1 are specific to conditions of stress. **A** Lifespan of *lea-1Δ* mutants is not significantly different from WT (N2) animals (*p* = 0.40, log-rank test). *lea-1(tm6452)* mutants were slightly short-lived relative to WT (N2) animals (*p* = 0.005, log-rank test). **B** Lifespan is not significantly altered by *lea-1Δ* or *lea-1(tm6452)* mutations in a *daf-2* mutant background (*p* = 0.11, *p* = 0.07, respectively, log-rank test). **C** There are no significant differences in brood size of *lea-1Δ* (*p* = 0.97, *n* = 3, unpaired *T* test) or *lea-1(tm6452)* mutants (*p* = 0.41, *n* = 3, unpaired *T* test) relative to N2 worms. Brood size of individual worms from independent replicates is shown. The mean is also shown and error bars represent 95% confidence intervals

Working with a null mutant allowed us to ask if there were any phenotypes associated with deletion of *lea-1* in the context of normal development and physiology. We tested effects on lifespan and brood size as especially sensitive quantitative proxies for diverse effects on physiology or development. Our results suggest that LEA-1 functions specifically in response to stress: In a wild-type (N2) background, we did not observe any significant differences in lifespan of *lea-1Δ* animals at 20 °C (Fig. 4A). We observed a modest reduction in lifespan of *lea-1(tm6452)* animals (*p* = 0.005), perhaps due to genetic background effects; the lack of reduction in lifespan in *lea-1Δ* animals demonstrates that complete loss of *lea-1* does not decrease lifespan. We also did not observe significant differences in polyglutamine aggregation during aging in these worms when maintained under normal laboratory conditions at 20 °C (Additional File 1: Fig. S4). Because LEA-1 expression is regulated by insulin-like signaling, we also tested for a difference in lifespan in the long-lived *daf-2* mutant background [47, 48]. We found that lifespans of *daf-2;lea-1Δ* and *daf-2;lea-1(tm6452)* were statistically indistinguishable from controls (Fig. 4B). Notably, there was higher mortality of *daf-2;lea-1(tm6452)* during the reproductive period, but this did not significantly influence maximum lifespan (Additional File 1: Table S2). The brood size of *lea-1Δ* and *lea-1(tm6452)* animals was also not significantly different from N2 controls (Fig. 4C). These results indicate that LEA-1 may function primarily during the dauer stage and in response to conditions of external stress.

### Expression of multiple LEA-1 isoforms is increased in response to desiccation

**Fig. 5:**
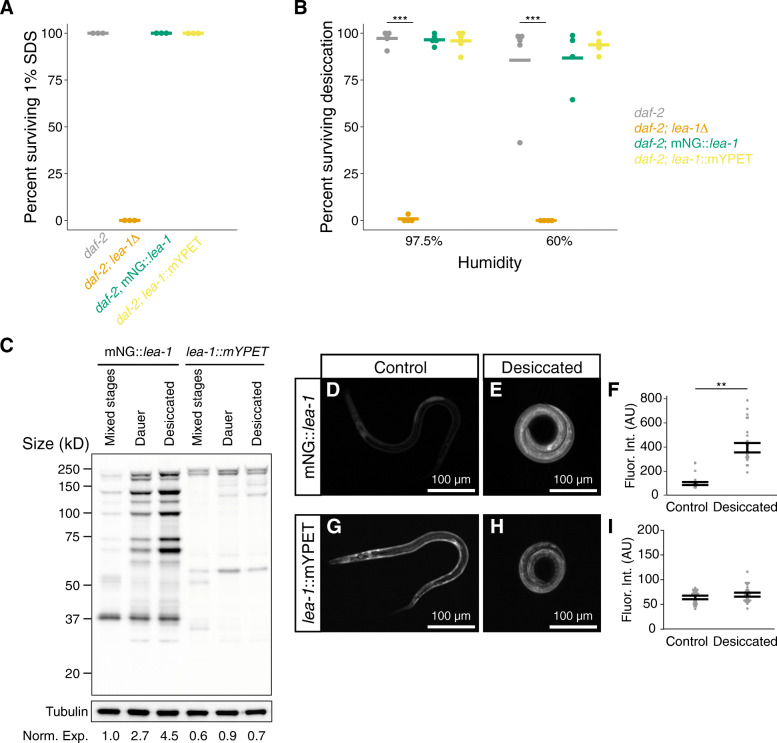
Functional endogenous fluorescent tags reveal increased LEA-1 protein expression of multiple isoforms during desiccation. **A** Endogenous fluorescent tags do not disrupt the function of LEA-1 in forming dauers that are resistant to 1% SDS (dauers formed at 25 °C). **B** Endogenous fluorescent tags do not disrupt the function of LEA-1 in desiccation survival. *lea-1Δ* are sensitive to desiccation at 97.5% relative humidity (*p* < 0.0001, *n* = 4, unpaired *T* test) and 60% relative humidity (*p* = 0.0002, *n* = 4, unpaired *T* test). Survival of mNeonGreen tagged LEA-1 worms (mNG::*lea-1*) and mYPET tagged worms (*lea-1*::mYPET) was not significantly different from *daf-2(e1370)* controls after exposure to 97.5% or 60% RH. **C** Visualization of protein samples extracted from mixed stage cultures, dauer worms, and desiccated worms and blotted with an anti-FLAG antibody indicates multiple isoforms tagged by the two independent tags. The membrane was stripped and blotted for tubulin as a loading control. Total protein in each lane was quantified and normalized to the tubulin loading control. Normalized proteins expression measurements, relative to mixed stage mNG::*lea-1* animals, are listed at the bottom of each lane (Norm. Exp.). **D** A representative dauer worm expressing mNG::*lea-1*. **E** A representative desiccated dauer larvae expressing mNG::*lea-1*. **F** mNG fluorescence is significantly increased in desiccated worms relative to non-desiccated controls (*p* = 0.001, *n* = 3 replicates with 14-15 worms per condition, unpaired *T* test). **G** A representative dauer worm expressing *lea-1*::mYPET. **H** A desiccated dauer expressing *lea-1*::mYPET. **I** Fluorescence intensity is not significantly different between control and desiccated dauer worms expressing *lea-1*::mYPET (*p* = 0.39, *n* = 3 replicates with 15 worms per condition, unpaired *T* test). Note that all worms were in a *daf-2(e1370)* background. **p < 0.01, ***p < 0.001

To examine where LEA-1 is expressed in vivo, we used fluorescent mNeonGreen (mNG) and monomeric yellow fluorescent protein for energy transfer (mYPET) tags to label endogenous LEA-1 at two different positions [49, 50]. Sequence for mNG as well as a 3x FLAG and auxin inducible degron sequence were inserted in the N-terminal region of the gene (Fig. 1). Similarly, sequence for mYPET and 3x FLAG were added to the C-terminus of LEA-1 (Fig. 1). As with the *lea-1Δ* mutant, these edits were confirmed via visualization of fluorescence and PCR (Additional File 1: Fig. S1E). Fusion of the fluorescent tags to LEA-1 did not disrupt resistance of dauer larvae formed at 25 °C to 1% SDS (Fig. 5A). When desiccated, animals expressing the fluorescent tags survived equally as well as *daf-2* control animals (Fig. 5B). We also confirmed that the fluorescent tags did not significantly impact osmotic stress survival in 1 M NaCl for 2 h (Additional File 1: Fig. S5A). We conclude that introduction of either fluorophore did not disrupt functions of the endogenous LEA-1 protein.

LEA-1 protein is known to be upregulated in response to desiccation [10, 30]. However, there are many predicted isoforms of LEA-1 (Fig. 1), and it is unclear whether all isoforms are upregulated or whether all of LEA-1’s protective roles involve such upregulation. To examine the diversity of isoforms that are actively expressed in different conditions we lysed cultures of mixed stage worms, dauer worms, and desiccated dauers to blot with an anti-FLAG antibody to recognize the 3x FLAG epitope appended to each fluorophore. Numerous isoforms appeared tagged with the 5′ mNG tag, while fewer isoforms of distinct sizes carrying the 3′ mYPET tag were prominent (Fig. 5C). Isoforms labeled with the 5′ mNG tag but not the 3′ mYPET tag were found to be upregulated during desiccation. These changes in expression were confirmed by quantification of in vivo fluorescence intensity in dauer and desiccated worms. Fluorescence from the mNG reporter was significantly increased after 4 days of desiccation at 97.5% relative humidity (Fig. 5D-F). Expression of mNG::LEA-1 was diffuse throughout the body during desiccation (Fig. 5E). In contrast, mYPET fluorescence level was not significantly altered during desiccation (Fig. 5G-I), consistent with the Western blot results. For both reporters, fluorescence intensity was not changed in response to 2 h of osmotic shock in 1 M NaCl (Additional File 1: Fig. S5B-G). We conclude that only some isoforms of LEA-1 are upregulated in response to desiccation and that *lea-1*’s response to osmotic shock does not appear to involve a similar upregulation.

### Depletion of LEA-1 in body wall muscle reduces survival of desiccation and osmotic stress

**Fig. 6:**
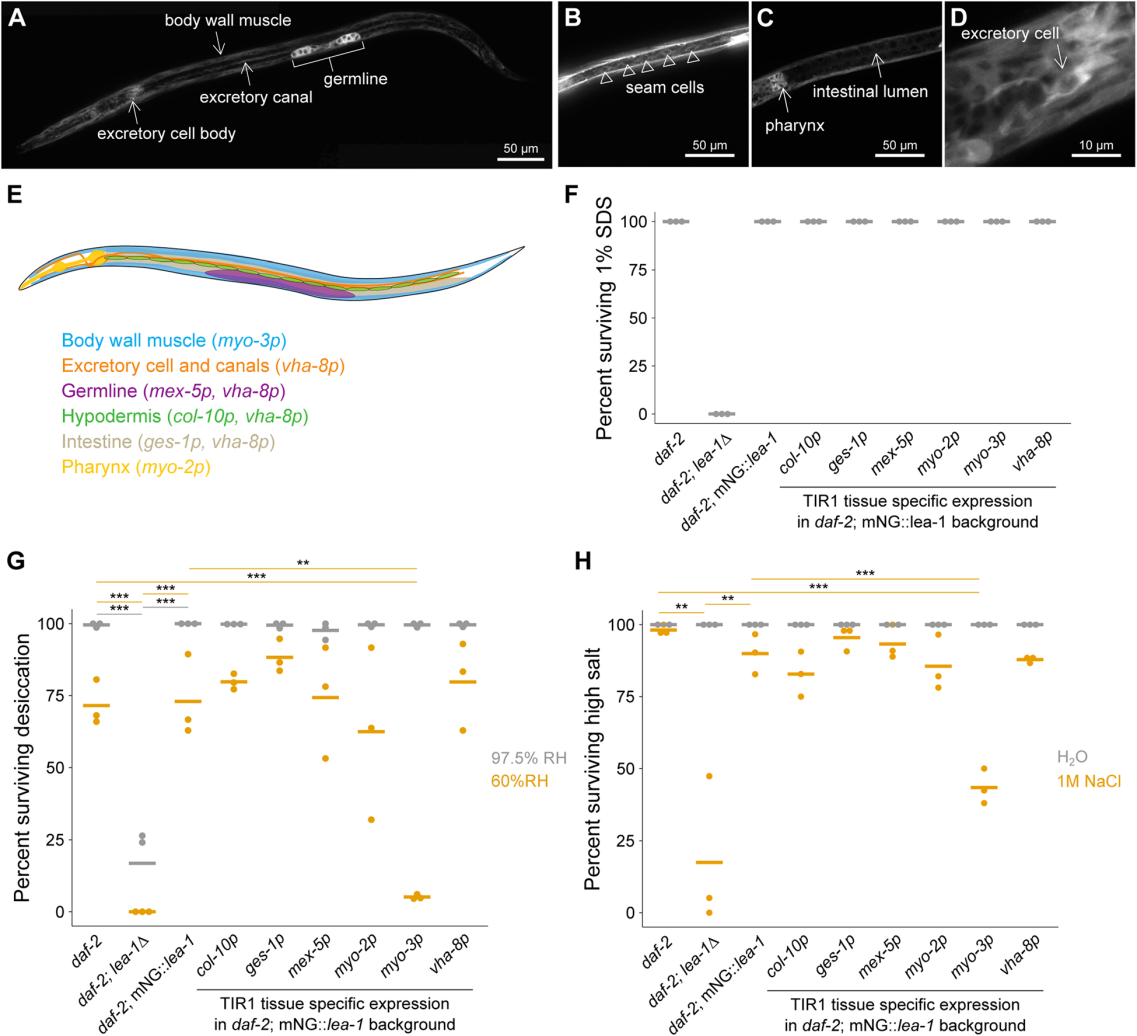
LEA-1 is required in body wall muscle to survive desiccation and osmotic stress. **A**–**D** Representative images of an mNG::*lea-1* worm depict the major sites of expression. There is prominent fluorescence in the germline, body wall muscle, excretory cell, and pharynx. There is also some apparent expression in seam cells (**B**) and faint fluorescence in the intestine (**C**). A zoomed in image of the excretory cell is shown in **D**. **E** A cartoon depicts tissues in which LEA-1 is expressed that were targeted for protein depletion by driving TIR1 under the control of different promoters. **F** Survival in 1% SDS is plotted for worms with tissue specific LEA-1 depletion by expression of TIR1 under various promoters and exposure to 1 mM auxin. Dauer larvae were formed at 25 °C. Depletion of LEA-1 did not sensitize worms to SDS. **G** Desiccation survival is plotted for worms with tissue-specific LEA-1. Depletion of the mNG::*lea-1* protein utilizing auxin-induced degradation reduces desiccation survival in worms expressing TIR1 under a *myo-3* (body wall muscle-specific) promoter relative to *daf-2* controls (*p* = 0.0001, *n* = 3, unpaired *T* test) and worms expressing only mNG::*lea-1* and not TIR1 (*p* = 0.001, *n* = 3, unpaired *T* test). No other site of TIR1 expression significantly altered desiccation survival at 60% RH. **H** Survival of osmotic stress in 1 M NaCl for 4 h is plotted for the same strains as in (**E**). *lea-1Δ* mutants and worms expressing TIR1 under a *myo-3* promoter were the only two strains that with significant differences in survival relative to both control and mNG::*lea-1*-expressing animals (*daf-2*;*lea-1Δ*: vs. *daf-2 p* = 0.006, vs. *daf-2;*mNG::*lea-1 p* = 0.01; *daf-2;*mNG::*lea-1;myo-3p*::TIR1: vs. *daf-2 p* = 0.0001, vs. *daf-2;*mNG::*lea-1 p* = 0.0009, unpaired *T* tests, *n* = 3). Note that the images in **A**–**D** show a worm with an N2 background, whereas worms in **F**–**H** are in a *daf-2(e1370)* background. **p < 0.01, ***p < 0.001

To examine the localization of upregulated LEA-1 isoforms in the stage when LEA-1 has a protective role, we visualized mNG::LEA-1 fluorescence in dauer larvae. We found that the protein was prominently expressed in specific tissues: the germline, body wall muscle, pharynx, and excretory cell (Fig. 6A–D). mNG::LEA-1 was localized to both the excretory cell body, which is adjacent to the pharynx (Fig. 6D), as well as excretory canals, which extend laterally along the length of the body (Fig. 6A). Some faint expression was seen in seam cells (lateral hypodermal cells) (Fig. 6B), as well as the intestine (Fig. 6C). Additionally, some unidentified cells in the head were fluorescent. The expression and localization of LEA-1 to particular tissues in dauer worms suggested that its function might be required in only some tissues or required at higher levels in some tissues than in others.

To determine the sites of action of LEA-1, we utilized auxin-induced degradation to deplete protein. We used the auxin-inducible degron (AID) sequence that we included on the mNG::3xFLAG tag as a target for protein degradation when the modified F-box protein TIR1 is expressed and worms are exposed to auxin [51]. Expression of TIR1 under the control of different promoters allows for tissue-specific conditional depletion [52]. We used a panel of worms expressing TIR1 in the various tissues in which LEA-1 was expressed to determine the tissue or tissues in which LEA-1 was required for survival of desiccation and osmotic stress (Fig. 6E) [52]. These experiments were conducted in a *daf-2* mutant background, for which expression patterns of LEA-1 were not noticeably different from a WT (N2) background (Additional File 1: Fig. S6A). When exposed to auxin, dauer worms showed depletion of mNG::LEA-1 in the expected locations for each promoter used: the intestine (using the *ges-1* promoter, called *ges-1*p), germline (*mex-5*p), pharynx (*myo-2*p), body wall muscle (*myo-3*p), and a combination of intestine, excretory cell and canal, germline, and some other cells in the head (*vha-8*p) (Additional File 1: Fig. S6). Expression of TIR1 with a *col-10* promoter to target hypodermis did not seem to have a significant effect on expression, consistent with the apparently minimal baseline hypodermal expression of mNG::LEA-1 (Additional File 1: Fig. S6C). Additionally, the *myo-2*p::TIR1 strain depleted some pharyngeal mNG::LEA-1 protein, but did not entirely eliminate expression, particularly in the posterior bulb of the pharynx (Additional File 1: Fig. S6F).

Depletion of LEA-1 in each of the TIR1 tissue specific strains tested did not disrupt the integrity of dauer larvae formed at 25 °C, as assessed by survival of 1% SDS (Fig. 6F). When exposed to desiccation at 97.5% relative humidity, LEA-1 depletion in TIR1 expression strains did not significantly reduce survival relative to *daf-2* or *daf-2;*mNG::*lea-1* animals (Fig. 6G). However, when exposed to 60% relative humidity, *myo-3*p::TIR1 worms had significantly reduced survival. This suggests that LEA-1 is required in the body wall muscle to promote desiccation resistance. When exposed to osmotic stress with 1 M NaCl for 4 h, we observed a similar result. *lea-1Δ* mutants and *myo-3*p::TIR1 worms had significantly reduced survival compared to *daf-2* and *daf-2;*mNG::*lea-1* worms (Fig. 6H). To determine if expression of LEA-1 in body wall muscle is sufficient for desiccation and osmotic stress survival, we attempted to rescue survival of *daf-2;lea-1Δ* mutants by expressing a *myo-3p::lea-1a::mNG* extrachromosomal array. Expression of LEA-1a in body wall muscle alone was not sufficient to restore SDS resistance and did not significantly improve tolerance of desiccation or osmotic stress in 1 M NaCl (Additional File 1: Fig. S7). Collectively, these results suggest that LEA-1 synthesized in the body wall muscle is necessary but not sufficient for survival of multiple stresses and that other tissues may not require high levels of the tagged LEA-1 isoforms for survival of animals.

### LEA-1 minimal motifs confer desiccation resistance to bacteria

**Fig. 7:**
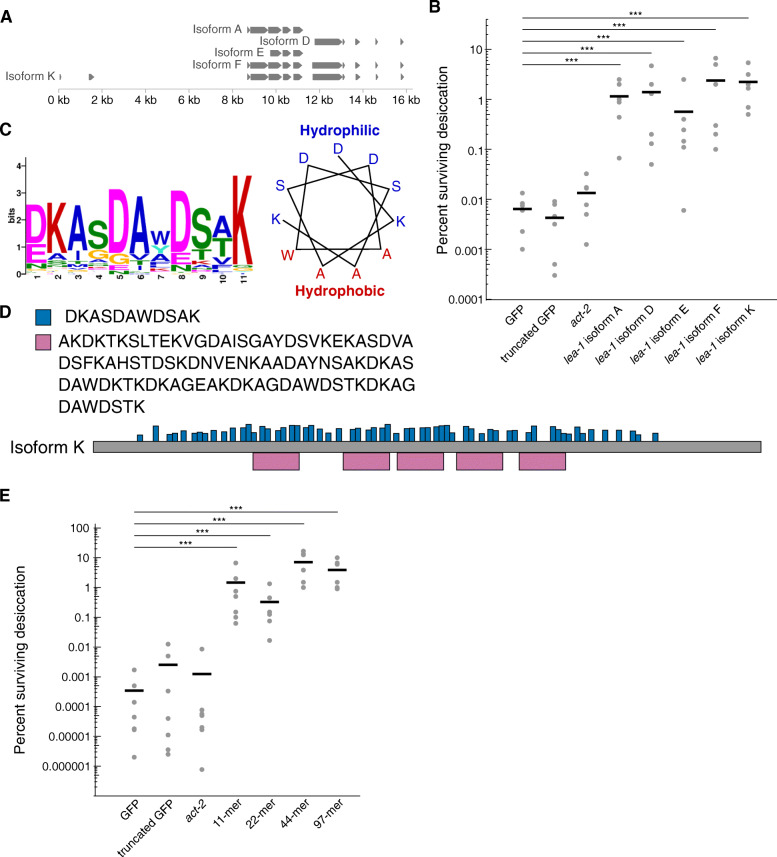
Heterologous expression of *C. elegans lea-1* isoforms and motifs improves bacterial desiccation survival. **A** Select isoforms of LEA-1 used for bacterial expression. **B** Desiccation survival of BL21 *E. coli* expressing codon-optimized *C. elegans* LEA-1 isoforms. Heterologous expression of each isoform increased desiccation survival (1-way ANOVA *p* < 0.0001, post-hoc Dunnett’s test: Isoform A *p* < 0.0001, Isoform D *p* < 0.0001, Isoform E *p* = 0.0008, Isoform F *p* < 0.0001, Isoform K *p* < 0.0001, each isoform compared to GFP control, *n* = 6). **C** A consensus 11-mer motif found in LEA-1 likely forms an amphipathic alpha helix. The position weight matrix of amino acids in the motif is shown, as well as a wheel diagram depicting the relative position of each consensus amino acid in an alpha helical conformation. **D** Consensus amino acid sequences are shown for the 11-mer as well as a 97-mer motif that was also detected with an expanded motif window size. The frequency and distribution of occurrences of these motifs within isoform K are shown. **E** Desiccation survival of BL21 *E. coli* is significantly increased by expression of motifs of LEA-1. Codon-optimized sequences for the 11-mer, as well as concatenated repeats of the 11-mer sequence (2x = 22-mer, 4x = 44-mer), and the 97-mer motif were expressed. Expression of each peptide increased survival of desiccated bacteria (1-way ANOVA *p* < 0.0001, post-hoc Dunnett’s test: 11-mer *p* < 0.0001, 22-mer *p* < 0.0001, 44-mer *p* < 0.0001, 97-mer *p* < 0.0001, compared to GFP control, *n* = 7). ***p < 0.001

Having described in vivo roles for an animal LEA protein and its localized expression and function, we sought to identify the protein’s protective domains. Given the variety of isoforms of *lea-1* in *C. elegans* that are expressed during desiccation (Fig. 1, Fig. 5C), before testing specific domains in vivo we employed a heterologous expression approach to efficiently test if different isoforms improved desiccation tolerance in *E. coli* equally or if some were more effective than others. We selected isoforms A, D, E, F, and K for expression because they have a combination of overlapping and non-overlapping sequence to allow for deduction of regions of interest in the case that some isoforms are more effective at conferring desiccation resistance (Fig. 7A). We confirmed robust expression of each of the 5 different bacterially codon-optimized isoforms in BL21 *E. coli* and found that expression of each isoform conferred stress resistance to the bacteria, relative to cells expressing GFP (Fig. 7B, Additional File 1: Fig. S8). Expression of a truncated version of GFP or actin (*C. elegans act-2*) did not increase desiccation resistance of bacteria (Fig. 7B).

Because all five isoforms tested were sufficient to improve bacterial desiccation resistance, we hypothesized that motifs shared by these isoforms might be sufficient for conferring bacterial desiccation resistance. Group 3 LEA proteins commonly have repeated motifs of 11 amino acids that are capable of protecting heterologous cells against stresses including low pH, high salt, and desiccation-induced protein aggregation [8, 21, 45, 53–56]. These motifs are predicted to form alpha helices, and circular dichroism of LEA proteins and motif-containing peptides has confirmed alpha helix formation during conditions when water is limiting [17, 18, 57]. We used MEME Suite to detect a repeated 11-mer from the protein sequence of one of the longest *C. elegans* isoforms, LEA-1 K [58]. An 11-mer was found that is predicted to form an amphipathic alpha-helix (Fig. 7C). When search parameters were relaxed to include motifs of other lengths, a 97-mer peptide encompassing multiple 11-mers was also identified. In the 1397 amino acid protein, there were five occurrences of the 97-mer and 61 instances of the 11-mer (Fig. 7D). To determine if these motifs alone from *C. elegans* LEA-1 were sufficient to improve desiccation tolerance, we expressed codon-optimized versions of these idealized motifs in *E. coli*. We included the base 11-mer, repeats containing two (22-mer) and four (44-mer) copies of its sequence, and the 97-mer. Expression of each of these minimal motifs significantly improved desiccation survival of the bacteria (Fig. 7E). Thus, it is likely that any of LEA-1’s isoforms that contain such repeats would improve survival of desiccation.

### LEA-1 motifs are sufficient for desiccation tolerance and osmotic stress survival in vivo

**Fig. 8:**
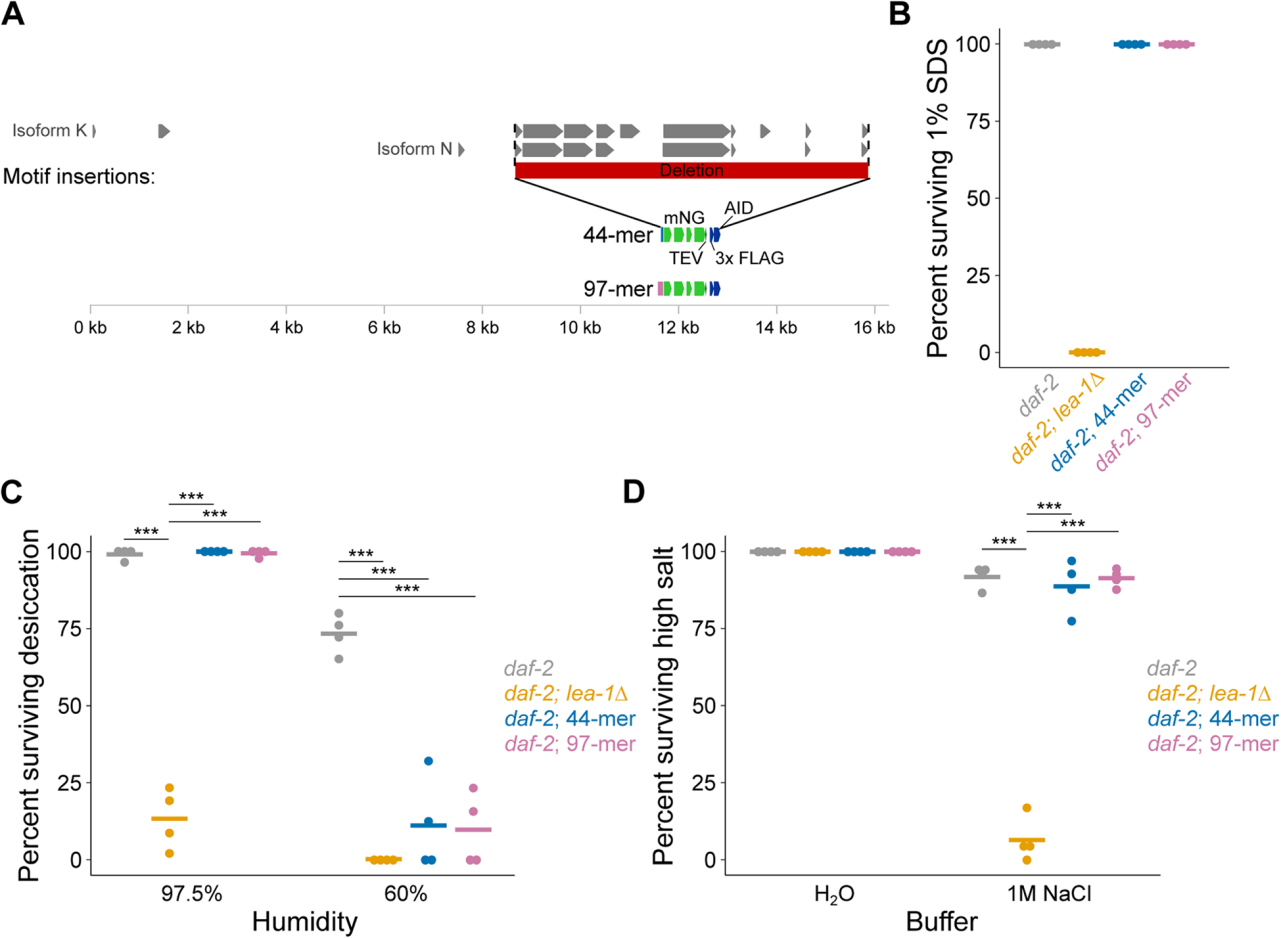
Minimal LEA-1 motifs promote desiccation and osmotic stress survival in vivo. **A** Endogenous *lea-1* sequence was largely deleted and replaced with sequence encoding codon optimized, mNG-tagged, idealized motifs of 44 amino acids (4 × 11-mer) or 97 amino acids. **B** Expression of 44 and 97 amino acid LEA-1 motifs is sufficient for the formation of dauer larvae (25 °C) that are resistant to 1% SDS. **C** Desiccation survival of *daf-2*, *daf-2;lea-1Δ*, *daf-2;*44-mer::mNG, and *daf-2;*97-mer::mNG animals is shown. *daf-2;lea-1Δ* mutants had significantly lower survival than control animals at both 97.5% RH (*p* < 0.0001, unpaired *T* test, *n* = 4) and 60% RH (*p* < 0.0001, unpaired *T* test, *n* = 4). At 97.5% RH motif-expressing worms had survival that was statistically indistinguishable from control and significantly improved relative to *daf-2;lea-1Δ* animals (44-mer *p* = 0.36 , 97-mer *p* = 0.78 , unpaired *T* tests, *n* = 4). When dehydrated at 60% RH, motif-expressing worms each had significantly reduced survival (*daf-2;*44-mer::mNG *p* = 0.0003, *daf-2;*97-mer::mNG *p* < 0.0001, unpaired *T* tests relative to *daf-2*, *n* = 4). **D** *daf-2;lea-1Δ* mutants, but not motif-expressing animals, were sensitive to osmotic stress in 1 M NaCl for 4 h (*daf-2;lea-1Δ p* < 0.0001, *daf-2;*44-mer::mNG *p* = 0.53, *daf-2;*97-mer::mNG *p* = 0.87 unpaired *T* test vs. *daf-2*, *n* = 4). Worms expressing LEA motifs had significantly improved survival relative to *daf-2;lea-1Δ* animals (*daf-2;*44-mer::mNG *p* < 0.0001, *daf-2;*97-mer::mNG *p* < 0.0001, unpaired *T* test, *n* = 4). ***p < 0.001

Because motifs of LEA-1 improved desiccation survival of bacteria, we were motivated to test if minimal motifs of LEA-1 are sufficient for stress tolerance in vivo. To specifically determine if minimal LEA-1 motifs are sufficient for desiccation tolerance in *C. elegans*, we deleted nearly all possible exons of *lea-1* and replaced them with sequence encoding single codon-optimized 44-mer or 97-mer motifs fused to mNG to allow visual confirmation of expression (Fig. 8A). Genomic sequence encoding the most N-terminal predicted exons for some isoforms (like the short N-terminal exons of isoforms K and N as shown in Fig. 8A) was left intact because these regions likely overlap with the promoter region or other regulatory sequence. These short predicted exons do not encode any 11-mer motifs. We confirmed genomic edits by PCR genotyping (Additional File 1: Fig. S9A,B). We also determined that the expression patterns of each inserted motif::mNG fusion are similar to the overall pattern observed for tagged versions of the endogenous protein, suggesting that these genomic edits did not significantly disrupt gene regulation (Additional File 1: Fig. S9C,D).

Expression of either the 44 amino acid motif or 97 amino acid motif was sufficient to replace full-length LEA-1’s role in dauer development. Each of the motif expression lines formed dauers at 25 °C that were resistant to 1% SDS (Fig. 8B). Expression of the 44-mer and 97-mer motifs also promoted desiccation tolerance of animals at 97.5% relative humidity (Fig. 8C). When exposed to 60% relative humidity, the protective capacity of these motifs was limited, and survival was reduced to levels comparable to *lea-1Δ* mutants. Expression of each motif in lieu of the complete LEA-1 protein was also sufficient to promote survival of osmotic stress in 1 M NaCl (Fig. 8D). Survival of motif-expressing animals was indistinguishable from controls and significantly improved relative to *lea-1Δ* mutants. Expression of these motifs specifically in body wall muscle was not sufficient to restore SDS resistance, desiccation tolerance, or osmotic stress survival to *daf-2;lea-1Δ* animals (Additional File 1: Fig. S9). The ability of short repetitive motif sequences under endogenous control to substitute for multiple functions of full-length LEA-1 suggests that these motifs may account for much of the functionality of the full-length LEA-1 protein during osmotic stress and desiccation.

## Discussion

LEA proteins are commonly found in animals with interesting extremotolerant abilities but with limited genetic tools. Therefore, a common approach has been to study LEA proteins (and fragments thereof) in heterologous systems [20, 21, 23, 26, 55, 56, 59–62]. While such approaches have revealed many potential functions of LEA proteins, there has been limited evidence to bridge protective abilities observed in vitro and in heterologous systems to the endogenous, in vivo context in an animal [10, 30, 63]. Therefore, we were motivated to use *C. elegans* as a model to test for in vivo functions of LEA-1 in its native context.

Previous studies of LEA-1 in *C. elegans* have often relied on RNAi phenotypes [10, 30]. We generated a null mutant (*lea-1Δ*) that lacks the entire gene, in order to eliminate the possibility of residual LEA-1 expression or function. Comparison of this mutant to the insertion and deletion mutant *lea-1(tm6452)* reveals similar sensitivities to a variety of stresses, including desiccation, osmotic stress, and heat. We also found that at 25 °C *lea-1* mutants formed dauer-like larvae that were sensitive to 1% SDS and had visible cuticle defects. The stress sensitive phenotypes assessed in dauer larvae were present even in SDS-resistant dauer larvae formed at 20 °C but in some cases were more severe in the dauer-like larvae formed at 25 °C. The cuticle limits the rate of water loss, so it is possible that the same defects that make *lea-1* mutants sensitive to SDS could allow more rapid loss of water and poorer desiccation survival. The function of multiple LEA-1 isoforms and amino acid motifs in improving desiccation tolerance of bacteria provides evidence that function of LEA-1 does not depend entirely on its role in dauer development, but likely functions as a protectant within cells during desiccation and related stresses.

We focused on measuring stress resistance in dauer larvae, in which LEA-1 is expressed more highly at both the level of mRNA and protein [10, 36]. DUR-1, the other group 3 LEA protein of *C. elegans*, is also upregulated in dauer larvae, but is apparently unable to compensate for the loss of LEA-1 [30]. LEA-1 does not significantly impact lifespan or brood size (Fig. 4), which demonstrates the importance of the protein specifically in the context of stress resistance. Similarly, accumulation of polyglutamine protein aggregates was exacerbated by deletion of LEA-1 in dauer worms, but not in aging adult worms (Fig. 3F, Additional File 1: Fig. S4). Effects of LEA-1 on protein aggregation have previously been demonstrated in vitro and in heterologous contexts [27, 43, 64]. We present evidence that LEA-1 can affect proteostasis in vivo in a multicellular animal. It would be fascinating to further explore any differential effects of LEA-1 with other types of aggregation-prone proteins and in tissues beyond the body wall muscle. Because LEA-1 is expressed highly in body wall muscle and is required there for desiccation survival and osmotic stress resistance, it is possible that improving proteostasis in this tissue contributes to survival of these stresses.

Adding endogenous, single-copy fluorescent tags to LEA-1 without disrupting LEA-1 function revealed the sites of its expression and in vivo dynamics in response to stress. In dauer larvae, mNG-tagged LEA-1 is expressed most notably in the germline, body wall muscle, pharynx, excretory cell, as well as some unidentified cells in the head. Expression patterns were similar in worms tagged with mYPET, although some sites of expression were more faint. Surprisingly, blotting for the 3x FLAG epitope included in both mNG and mYPET tags revealed significantly fewer isoforms tagged by mYPET than expected based on isoform predictions if all isoforms were expressed at high levels (Fig. 1, Fig. 5C). It is possible that the C-terminal exons are not actually included in as many isoforms as expected or that some isoforms are expressed at low levels. Another possibility is that LEA-1 protein is processed by cleavage into shorter peptides [65]. The difference in the number of prominent isoforms labeled by each tag is further reflected in the significant increase in expression of mNG-labeled proteins but not mYPET-labeled proteins during desiccation (Fig. 5C–I). The upregulation of mNG-tagged proteins does not seem to be specific to a single isoform, but rather, multiple isoforms are expressed more highly during desiccation (Fig. 5C). Therefore, the increased fluorescence seen in vivo likely reflects the summation of these isoforms of varying sizes and compositions. LEA-1 has been reported to be regulated by transcription factors including *daf-16*/FOXO and *skn-1*/Nrf, but whether such regulation is tissue-specific or unique to some isoforms remains unexplored [47, 66]. Expression of LEA-1 in several tissues, and the upregulation of multiple isoforms led to two questions: (1) In which tissue or tissues is LEA-1 required for stress resistance? (2) Are particular isoforms more effective at conferring stress resistance?

To address the first question, we utilized the auxin-inducible degradation system to deplete LEA-1 in various tissues [52]. By expressing TIR1 under the control of different promoters, we had control over the tissues in which proteins containing the auxin-inducible degron (AID) tag were degraded. The AID sequence was included on the mNG tag and therefore should deplete the same isoforms containing the 3x FLAG tag (Fig. 5C). It is possible that some predicted isoforms that are not tagged with the mNG construct could escape degradation (for example isoforms C, D, and E, Fig. 1), although we do not know if such isoforms are expressed or their relative abundance. Furthermore, even though we observed decreased fluorescence in most cases (Additional File 1: Fig. S6), depletion of AID-containing proteins may be incomplete. Incomplete degradation could result from insufficient levels of TIR1 (based on the strength of the promoter driving it) in tissues with high levels of LEA-1. For example, with *myo-2p* driven TIR1, some expression of mNG::LEA-1 remained in the pharynx—particularly in the posterior bulb (Fig S6F). Thus, we cannot rule out the possibility that LEA-1 is required in the pharynx for survival of desiccation and osmotic stress. In contrast, depletion of LEA-1 in body wall muscle resulted in significantly reduced survival of both desiccation and osmotic stress (Fig. 6G, H). The consensus site of action from these two stresses lends confidence to the result. However, given the caveats that some untagged isoforms may remain, and that there could be incomplete degradation (even if fluorescence is depleted below detectable levels), we cannot formally conclude that LEA-1 is not required in other tissues. Rather, we present evidence that LEA-1 is required at high levels in at least body wall muscle to promote desiccation and osmotic stress tolerance. Yet, expression in muscle alone is insufficient for stress resistance (Additional File 1: Fig. S7, Additional File 1: Fig. S10). Overall, our data suggest that other tissues can survive at least with reduced levels of the LEA-1 isoforms that are induced in dauer larvae. For robust survival during desiccation and osmotic stress, LEA-1 is necessary in body wall muscle but also likely required in some combination of other tissues.

There are several possible explanations for the relative importance of LEA-1 in body wall muscle. It is possible that the location of body wall muscle, which covers a significant area along the circumference of dauer larvae, could function as a sub-cuticular sheath to protect the animal [67]. However, it is also conceivable that the absolute abundance of LEA-1 rather than a particular tissue-specific distribution is essential for survival. Because body wall muscle is a large tissue and expresses LEA-1 at high levels, depletion from this site of production could more significantly deplete the overall pool of LEA-1 in the animal. A third possibility is that decreased proteostasis in body wall muscle (as suggested by protein aggregation in Fig. 3F) could lead to paralysis and death.

To answer the second question of which LEA-1 isoforms are most functional in conferring stress resistance, we initially employed a heterologous expression approach. *E. coli* provides an ideal system to facilitate expression of proteins and rapidly screen for desiccation resistance. The fact that multiple LEA-1 isoforms could improve desiccation tolerance of bacteria turned our attention to conserved motifs contained within these isoforms. Similar motifs from LEA proteins of other organisms have been demonstrated to function in stress resistance [21, 45, 55, 56, 68]. These LEA motifs acquire secondary structure during desiccation-like conditions and specifically are thought to form amphipathic alpha-helices [19, 57, 69]. The 11-mer we identified is likely to form an amphipathic helix based on the amino acid composition and locations as depicted in Fig. 7C. Although the amino acid identity is different from other similarly identified LEA motifs, the putative structural similarities and charge distribution hint at the relative importance of these biochemical properties over sequence identity [12, 69]. Expression of the 11-mer alone was sufficient to improve bacterial desiccation survival. Increased numbers of motif repeats may improve survival, although the importance of multiple motifs occurring in the same contiguous peptide versus the possibility of oligomerization remains unclear. Notably, in bacteria, the expression levels of LEA proteins and motifs are likely much higher than in endogenous contexts.

Inserting motifs into the endogenous *lea-1* locus of *C. elegans* allowed us to test if the identified motifs are sufficient to confer desiccation in vivo. LEA motifs were highly effective substitutes for the full-length protein for phenotypes of osmotic stress resistance and desiccation at 97.5% RH (Fig. 8). This suggests that the function of LEA-1 can in large part be explained by the presence of these amino acid repeats. The inability of these motifs to fully rescue desiccation survival at 60% humidity could be a result of the appended fluorescent tag disrupting some aspect of the motif’s function. It is also possible that larger order repeats are necessary during more severe desiccation. Unique combinations of motifs or longer contiguous sequences may allow for increased functionality—particularly in the context of more severe desiccation. This may further explain the apparent complexity and variety of isoforms of the protein (Fig. 1, Fig. 5C). If there is limited selective pressure for maintenance of a single long contiguous sequence, then many functional exon combinations may arise. This could contribute to the large number of proteins produced by this gene. An optimal number of motifs and the most functional combination of motif variants appended to each other remain to be characterized.

The ability of LEA motifs to function similarly in a single-celled prokaryote (*E. coli*) and a multicellular eukaryote (*C. elegans*) encourages the possibility of engineering broadly functional desicco-protectants. LEA proteins and motifs that can function in cells with fundamentally different subcellular organization likely harbor basic biochemical properties that promote cell and organismal survival. Relative to bacteria, multicellular animals face further challenges during desiccation including coordination of a response across multiple tissue types and protecting diverse organelles and subcellular compartments. *C. elegans* is a prime animal model for continued study of these in vivo mechanisms of desiccation tolerance.

## Conclusions

In summary, we created a null mutant of LEA-1 to study its role in vivo and characterized a temperature-sensitive dauer formation defect and several phenotypes of stress sensitivity. LEA-1 is expressed in multiple tissues, and it is required at high levels in body wall muscle to carry out its functions. We identified LEA-1 motifs that are sufficient to improve bacterial desiccation survival. These motifs also function in place of full-length LEA-1 to promote desiccation tolerance in vivo. Future work is needed to dissect the principles by which the peptide repeat sequences function. Still, the conserved function of LEA-based peptides in a single-celled prokaryote and a multicellular animal suggests the possibility of engineering LEA-1-derived peptides that broadly promote desiccation tolerance.

## Methods

### CRISPR editing and generation of extrachromosomal array lines in C. elegans

To create a deletion of *lea-1* and to insert mNG and mYPET fluorescent tags into the endogenous gene locus, CRISPR methods were employed [34, 35]. Using the self-excising cassette (SEC) system, sgRNA sequences were added to the sgRNA-Cas9 plasmid pDD162 using the NEB Q5 site-directed mutagenesis kit. Homology arms of ~ 500–700 bp were added to plasmids carrying the SEC and repair templates. To insert a myo-2p::GFP::myo-2 3′UTR reporter to track the null deletion of *lea-1*, homology arms were cloned into plasmid pDD317. To insert mNG or mPYET tags, homology arms were cloned into pUA77 and pDD283 respectively.

To replace the endogenous *lea-1* sequence with LEA motifs, sequence was first codon-optimized for *C. elegans* and synthesized by Integrated DNA Technologies (IDT). These stretches were cloned into pUA77 with the same upstream homology arm for insertion of mNG to the endogenous locus and the same downstream homology arm for insertion of mYPET into the endogenous locus. The same sgRNAs for each of the initial mNG and mYPET insertions were used in combination to excise the *lea-1* locus.

Worms were injected with 50 ng/μL of plasmid containing the sgRNA and Cas9, and 10–20 ng/μL plasmid containing the repair template, along with a co-injection mix [35]. Two to three days after injection, worms were treated with Hygromycin and selected for transgene-carrying rollers lacking red co-injection mix extra-chromosomal arrays. L1 worms were heat shocked at 32 °C for 5 h to excise the SEC. Genomic edits were confirmed by visualization of fluorescent reporters and with PCR genotyping.

Construction of most TIR1-expressing strains is described in [52]. The *col-10p*::TIR1 line was generated by cloning to combine the *col-10* promoter with the TIR1 construct of pDD356 (NEB Hifi Assembly Master Mix). Plasmid pAP082 was used to express Cas9 and a sgRNA to target the chromosome I insertion site. Worms were injected with 50 ng/μL of pAP082 and 20 ng/μL plasmid containing the repair template.

To establish extrachromosomal array lines expressing *lea-1a* or motifs specifically in body wall muscle, the *myo-3* promoter and an mNG fluorescent tag was cloned onto each of *lea-1* isoform A, a 44-mer motif sequence, and a 97-mer motif sequence. DNA was injected into the gonads of LP852 *daf-2(e1370);lea-1Δ(cp423[myo-2p::GFP::myo-2 3′UTR])* animals at a concentration of 50 ng/μL. Progeny reliably expressing and transmitting the mNG labeled construct in body wall muscle were selected and established as transgenic array lines.

### Genotyping

Template genomic DNA was create by lysing worms in lysis buffer with proteinase K. Worms were picked into 0.2 mL tubes and briefly frozen at − 80 °C, then heated at 65 °C for 1 h followed by 95 °C for 15 min. This lysate was used for PCR genotyping. Primers were designed to confirm CRISPR genome modifications and to genotype the pre-existing allele *lea-1(tm6452)* during backcrossing. Sequences of these primers can be found in Additional File 1: Supplemental Table 1. PCR genotyping was carried out using either Gotaq or Q5 High Fidelity Polymerase (NEB). Annealing temperature and extension time were adjusted based on the melting temperature of the primers and size of the amplicon.

### C. elegans maintenance

Worms were maintained according to standard laboratory conditions on nematode growth media (NGM) plates, fed OP50 *E. coli*, and stored at 20 °C. Standard methods were employed for crossing worms, and males were generated by keeping L4 worms at 32 °C for 5–6 h and 25 °C overnight. The following strains and alleles were used: N2, *lea-1(tm6452)* (backcrossed 5x into N2), AM140 *rmIs132[unc-54p::Q35::YFP]*, AM141 *rmIs133[unc-54p::Q40::YFP]*, CB1370 *daf-2(e1370)*, LP847 *lea-1Δ(cp423[myo-2p::GFP::myo-2 3′UTR])*, LP852 *daf-2(e1370);lea-1Δ(cp423[myo-2p::GFP::myo-2 3′UTR])*, LP858 *lea-1(cp431[mNG*::*3x FLAG::AID::lea-1])*, LP859 *lea-1(cp430[lea-1::mYPET::3x FLAG])*, LP860 *daf-2(e1370)*;*lea-1(cp431[mNG*::*3x FLAG::AID::lea-1])*, LP861 *daf-2(e1370)*;*lea-1(cp430[lea-1::mYPET::3x FLAG])*, LP862 *rmIs132[unc-54p::Q35::YFP];lea-1Δ(cp423[myo-2p::GFP::myo-2 3′UTR])*, LP863 *rmIs133[unc-54p::Q40::YFP];lea-1Δ(cp423[myo-2p::GFP::myo-2 3′UTR])*, LP865 cpSi171[vha-8p::TIR1::F2A::mTagBFP::AID*::NLS];daf-2(e1370);lea-1(cp431[mNG::3x FLAG::AID*::lea-1]), LP866 cpSi172*[myo-2p::TIR1::F2A::mTagBFP::AID::NLS];daf-2(e1370);lea-1(cp431[mNG*::3x FLAG::AID*::lea-1]), LP867 cpSi173[col-10p::TIR1::F2A::mTagBFP::AID*::NLS];daf-2(e1370);lea-1(cp431[mNG::*3x FLAG::AID::lea-1])*, LP868 cpSi174[myo-3p::TIR1::F2A::mTagBFP::AID*::NLS];daf-2(e1370);lea-1(cp431[mNG::*3x FLAG::AID::lea-1])*, LP875 *daf-2(e1370);lea-1(tm6452, 5x backcrossed)*, LP876 reSi5[ges-1p::TIR1::F2A::BFP::AID*::NLS::tbb-2 3′UTR];daf-2(e1370);lea-1(cp431[mNG::*3x FLAG::AID::lea-1])*, LP877 wrdSi18[mex-5p::TIR1::F2A::BFP::AID*::NLS::tbb-2 3′UTR];daf-2(e1370);lea-1(cp431[mNG::*3x FLAG::AID::lea-1])*, LP882 *rmIs132[unc-54p::Q35::YFP]*;*daf-2(e1370);lea-1Δ(cp423[myo-2p::GFP::myo-2 3′UTR])*, LP883 *rmIs133[unc-54p::Q40::YFP];daf-2(e1370);lea-1Δ(cp423[myo-2p::GFP::myo-2 3′UTR])*, LP885 *daf-2(e1370);lea-1(cp432[lea-1p::44-mer::mNG::3x FLAG::AID]*, LP887 *daf-2(e1370);lea-1(cp433[lea-1p::97-mer::mNG::3x FLAG::AID]*.

### Analysis of dauer larvae

To assess SDS resistance of larvae we picked dauer or dauer-like larvae into 1 mL of 1% SDS. After 15 min, worms were scored for viability by movement in response to gentle prodding.

To determine if worms were actively feeding, we employed a fluorescent bead-eating assay [70]. Cultures of *E. coli* OP50 were grown overnight at 37 °C with shaking, and 10 mL of culture were centrifuged at 5000 rpm for 10 min and resuspended in 1 mL LB. Plates were prepared by adding 1 μL of red fluorescent latex beads (Sigma L3280) to the concentrated bacteria and plating ~ 60 μL onto NGM plates. Worms were added to the lawns of bacteria on dry plates and allowed to feed for 30 min. The number of worms with ingested beads was scored by visual inspection using a Zeiss Axio Zoom.V16 to identify red fluorescence in the pharynx or intestine of each worm.

Representative images of larvae were taken using DIC optics on a Nikon Eclipse E800 microscope and a pco.panda sCMOS camera. To calculate the width of larvae as a measure of radial constriction, images were analyzed in FIJI. The width of worms was measured immediately posterior to the pharyngeal bulb.

### Scanning electron microscopy

*C. elegans* were fixed in 2% paraformaldehyde/2.5% glutaraldehyde in 0.15 M sodium phosphate buffer, pH 7.4, for 1 h at room temperature and stored at 4 °C. Following three washes in 0.15 M sodium phosphate buffer, pH 7.4, the samples were post-fixed in 1% osmium tetroxide in 0.15 M sodium phosphate buffer, pH 7.4 for 1 h and gently washed three times in deionized water. Samples were then deposited onto a poly d lysine coverslip or transferred to a microporous chamber and gradually dehydrated with ethanol (30%, 50%, 75%, 100%, 100%). Coverslips and microporous capsules were then transferred to a Samdri-795 critical point dryer and dried using carbon dioxide as the transitional solvent (Tousimis Research Corporation, Rockville, MD). Samples were mounted on 13 mm aluminum stubs with double-sided carbon adhesive and sputter coated with 5 nm of gold-palladium alloy (60 Au:40 Pd, Cressington Sputter Coater 208HR, model 8000-220, Ted Pella, Redding, CA). Specimens were observed and images taken using a Zeiss Supra 25 FESEM operating at 5 kV, using the SE2 detector, 5 mm working distance, and 30 μm aperture (Carl Zeiss SMT Inc., Peabody, MA).

### C. elegans desiccation, heat, and osmotic stress

*C. elegans* were desiccated according to a previously established protocol [30, 36]. Briefly, desiccation chambers were made with varying ratios of glycerol to water in order to produce relative humidity (RH) of either 97.5% or 60% [71]. Dauer worms were formed by moving *daf-2(e1370)* mutant embryos to 25 °C. Dauer worms were picked into ~ 1.5–2 μL droplets of water and initially exposed to 97.5% for 4 days as preconditioning. Worms were either rehydrated in M9 or moved to 60% RH desiccation chambers for 1 day before recovery. Survival was scored as the percentage of worms moving or responsive to physical stimulus.

Heat stress was implemented by exposing dauer worms to 37 °C. Multiple plates containing dauer larvae were moved to a 37 °C incubator for defined periods of time. Worms were scored for survival based on movement or responsiveness to physical stimulus.

To measure osmotic stress tolerance, dauer worms were added to water or 1 M NaCl in a 96-well or 24-well plate for 2 h or 4 h. Worms were then transferred to unseeded NGM plates and survival was scored by movement or responsiveness to physical stimulus.

### C. elegans lifespan and brood size

For lifespan assays, ten L1 worms per plate were picked to an NGM plate. For each genotype, five plates of ten worms were included to start each experiment. Worms were maintained at 20 °C and transferred to new plates every other day until they were no longer reproductive. Worms that crawled onto the sides of the dishes or were otherwise missing were censored. Survival was scored every other day by movement or responsiveness to touch with a platinum wire. Three independent biological replicates were conducted.

To determine brood size, individual embryos were added to NGM plates. Fifteen worms were included to start in each of three experiments. Worms were transferred to new plates every other day. After transfer, progeny on plates were allowed to develop for ~ 24 h and were then counted. Total brood was calculated as the sum of all progeny produced by an individual worm. Worms that failed to hatch or crawled off the plate were censored.

### Polyglutamine protein aggregation

Strains AM140 and AM141 express polyglutamine tracts of 35 or 40 repeated glutamine residues fused to yellow fluorescent protein (YFP) [46]. These strains were crossed with *lea-1Δ* (LP847). To assess protein aggregation during aging the worms were picked to plates and allowed to develop for 4 or 8 days. Worms were periodically transferred to fresh plates to separate them from progeny. The number of aggregates per worm was scored by imaging worms and counting the number of fluorescent puncta in the body wall muscle of each worm.

To determine the number of aggregates in dauer worms the AM140 and AM141 reporters were crossed into *daf-2(e1370);lea-1Δ* (LP852) to put the reporter in a *daf-2* mutant background. Dauer formation was induced as before by growth at 25 °C. The number of aggregates per worm was counted after 5 days (including development and arrest as dauer larvae).

### Fluorescent imaging and quantification

Worms expressing mNG::LEA-1 or LEA-1::mYPET were imaged on a Nikon TiE stand with CSU-X1 spinning disk head (Yokogawa), 514 nm solid state laser, and ImagEM EMCCD camera (Hamamatsu). Metamorph software was used for image acquisition. To quantify fluorescence in control dauers, desiccated dauers, and dauers exposed to 1 M NaCl for 2 h, worms were imaged with a × 10 objective. Images were imported into FIJI for analysis. Whole worms were outlined manually. The total fluorescence intensity was measured. The outline of the worm was moved to background area of the image to obtain a background measurement. The fluorescence intensity was calculated by subtracting the background from that of the worm. For representative images of worms higher magnification objectives (× 20 or × 60) were used.

### Western blotting

Protein for western blotting was obtained from worms expressing mNG::3x FLAG::LEA-1 or LEA-1::mYPET::3x FLAG. Mixed stage populations were washed from 3 to 6 NGM plates, dauer worms from plates at 25 °C, and desiccated worms from plastic dishes at 97.5% RH for 4 days. Large quantities (> 20,000) of synchronous worms were obtained for dauer formation and desiccation by standard hypochlorite treatment. Worms were collected, washed in M9, pelleted, and resuspended in ~ 100 μL lysis buffer containing 50 mM HEPES (pH 7.4), 1 mM EGTA, 1 mM MgCl_2_, 100 mM KCl, 10% glycerol, 0.05% NP-40, DTT, and an EDTA-free protease inhibitor tablet (added fresh to 12 mL of buffer) [72]. Worms in lysis buffer were briefly frozen at − 80 °C, then sonicated on ice with a Branson Sonifier 250 wand sonicator at 50% duty until no intact worms remained (2–5 min in individual bouts of no longer than 1–2 min). The concentration of protein in lysates was determined with the Biorad Protein Assay (Bradford) according to specifications, measuring the OD595 in a total volume of 1 mL in cuvettes.

A 4–12% Bis-Tris NuPAGE minigel (Invitrogen), loaded with 10 μg of protein in sample buffer (3x concentrate contains 6% SDS, 240 mM Tris pH 6.8, 30% glycerol, 0.04% w/v Bromophenol blue, and 50 μL 2-ME per mL) was run in 1x NuPAGE MOPS running buffer (Novex). Ten microliters of Precision Plus Protein Kaleidoscope Standard (Biorad) was loaded in lanes adjacent to samples. Gels were run at 140 V for 90 min. Protein was transferred to a PVDF membrane activated in methanol at 90 V for 90 min (in NuPAGE transfer buffer, at 4 °C). Membranes were rinsed in PBST, and blocked in PBST + 5% BSA for 1 h with rocking. Membranes were soaked in a 1:1000 dilution of primary antibody (mouse anti-FLAG, Sigma Cat #F1804) overnight at 4 °C with rocking. Membranes were washed 3x in PBST, then soaked in a 1:5000 dilution of secondary antibody (goat anti-mouse IgG, Invitrogen Cat #31432) for 1 h with rocking. Membranes were washed 3 times and visualized after addition of SuperSignal West Pico PLUS Chemiluminescent Substrate (Thermo Scientific).

After initial visualization, membranes were stripped and re-blotted for tubulin as a loading control. Membranes were incubated in mild stripping buffer (15 g glycine, 1 g SDS, 10 mL Tween20, bring volume to 1 L with H_2_0, pH 2.2) for 10 min with rocking. Buffer was replaced, and allowed to soak for another 10 min. Membranes were then washed 3x in PBST, blocked for 1 h in PBST with 5% BSA, and soaked in a 1:500 dilution of primary antibody (rat anti-tubulin, Invitrogen Cat #MA1-80189) overnight at 4 °C. After washing 3x in PBST, secondary antibody (goat anti-rat IgG, Thermo Fisher Cat #31470) was added in a 1:5000 dilution and membranes soaked for 1 h with rocking and visualized as before. A single band was detected indicating antibody specificity for tubulin.

### Protein depletion with auxin-induced degradation

Worms expressing TIR1 under various promoters were grown on plates containing 1 mM auxin (indole-3-acetic acid) [51]. Embryos or L1s were picked to NGM + auxin plates and allowed to develop into dauer larvae at 25 °C for at least 5 days before exposure to desiccation or osmotic stress. Degradation of LEA-1 tagged with mNG::3x FLAG::AID was assessed by imaging worms.

### Heterologous expression in bacteria

A bacterial codon-optimized version of *lea-1* isoform K was synthesized by Genewiz. Isoforms A, D, E, and F were subcloned from that construct; 30 bp of homology was added by PCR to expression constructs, and they were cloned into a PCR-linearized pDest17 using NEB Hifi Assembly Master Mix. Similarly, to clone motifs, 30 bp homology was added synthesized codon-optimized DNA fragments (from Integrated DNA Technologies), and they were cloned into PCR-linearized pDest17 with NEB HiFi Assembly Master Mix. GFP and truncated GFP controls were cloned by the same method, using plasmid pFCcGi as a template to obtain the GFP sequence [73]. The *C. elegans act-2* gene was also cloned as a control from cDNA. All plasmids were transformed into NEB 5-alpha cells and correct inserts were verified by sequencing.

For desiccation experiments, plasmids containing expression constructs were transformed into *E. coli* BL21 AI (Invitrogen). Individual colonies were picked into 3 mL LB with 100 μg/mL ampicillin (Amp) and grown overnight (~ 12–16 h) at 37 °C in a shaker incubator. Cultures were then diluted 1:20 and 0.2% L-arabinose was added to induce protein expression for 4 h. Protein expression was confirmed with a Coomassie gel. Bacteria were pelleted and briefly boiled to generate a lysate. Protein concentration was quantified using the Biorad Protein Assay (Bradford) according to specifications. From these protein concentrations, an appropriate volume was determined to load 20 μg of total protein per well. Five microliters of pageruler prestained ladder was loaded. A 4–12% BT NuPAGE minigel was run in 1x NuPAGE MOPS running buffer. The gel was stained with Coomassie and de-stained overnight. GFP-expressing bacteria provide a convenient visual confirmation of protein expression in each experiment.

To obtain equivalent numbers of starting bacteria to measure desiccation survival, OD600 of 4 h induced cultures was measured and volumes equivalent to an OD600 of 1.5 were added to 1.5 mL Eppendorf tubes. Bacteria were washed once in 0.85% NaCl. Bacteria were then resuspended in 1 mL 0.85% NaCl. A 20 μL sample was removed and used for 10-fold dilution of the culture. A 10-fold dilution series was plated on LB + Amp agar plates by spotting 10 μL. These control plates were grown at 37 °C overnight. The remaining 980 μL supernatant was aspirated and tubes were desiccated in a speedvac overnight (~ 12–16 h). Bacteria were rehydrated in 980 μL of LB + Amp and given 1–2 h to recover before plating a 10-fold dilution series, as with controls the day before. Bacteria on plates were grown overnight at 37 °C. Survival was calculated by dividing cfu of each desiccated sample by its control.

### Motif analysis

The MEME tool of the MEME suite (http://meme-suite.org/tools/meme) was used to identify conserved motifs within LEA-1 [58]. The protein sequence of Isoform K was used as a template since it contains nearly all possible exons of the protein. Position weight matrices, consensus motifs, and distribution of motifs throughout the protein were obtained from this analysis. The pepwheel program (https://www.bioinformatics.nl/cgi-bin/emboss/pepwheel) was used to generate the diagram of the putative alpha-helical structure formed by the 11-mer. Coloration of amino acids was added by hand.

### Statistical analysis

Statistical tests were carried out as described in figure legends. Unpaired t-tests were conducted in Microsoft Excel. ANOVAs and post-hoc Dunnett’s tests were conducted in RStudio. For analysis of bacterial desiccation survival, percent survival was log transformed to standardize variance for statistical analysis. Variance was assessed with Bartlett’s test. Lifespan analysis was conducted on aggregate data from three biological replicates with OASIS 2, and data were plotted with GraphPad Prism [74].

## Supplementary Information

**Additional file 1: Figure S1.** Genotyping LEA-1 alleles. **Figure S2.** Representative images of dauer-like larvae of *lea-1* mutants. **Figure S3.** Mutations in *lea-1* impact wild-type worms (N2) similarly to *daf-2* mutants. **Figure S4.** LEA-1 does not significantly alter polyglutamine protein aggregation due to age. **Figure S5.** LEA-1 expression does not increase in response to short-term osmotic stress. **Figure S6.** Representative images of auxin-induced depletion of mNG::*lea-1* in TIR1 expressing strains. **Figure S7.** Overexpression of *lea-1a* in body wall muscle does not rescue SDS sensitivity, desiccation tolerance, or osmotic stress resistance. **Figure S8.** Controls for heterologous expression of proteins in bacteria. **Figure S9.** Characterization of LEA-1 motif-expressing worms. **Figure S10.** Expression of LEA-1 motifs in body wall muscle does not improve SDS resistance, desiccation tolerance, or osmotic stress survival. **Table S1.** Genotyping primers for lea-1 genomic edits. **Table S2.** Lifespan analysis of *lea-1* mutants.

**Additional file 2.** Raw data.

## Data Availability

All data generated or analyzed during this study are included in this published article and supplementary information files. Raw data are included in Additional File 2. *C. elegans* strains generated in this study will be deposited to the *Caenorhabditis Genetics Center*. Plasmids and other reagents will be made available upon request.

## Abbreviations

AID: Auxin-inducible degron
GFP: Green fluorescent protein
LEA: Late embryogenesis abundant
mNG: mNeonGreen
mYPET: Monomeric yellow fluorescent protein for energy transfer
SEC: Self-excising cassette
YFP: Yellow fluorescent protein
