# Supplementary material for: LEA motifs promote desiccation tolerance in vivo
Source: BMC Biol. 2021 Dec 14;19:263. doi: 10.1186/s12915-021-01176-0 (PMC8670023; doi:10.1186/s12915-021-01176-0)
Supplement: Supplementary file 1 — Additional file 1: Figure S1. Genotyping LEA-1 alleles. Figure S2. Representative images of dauer-like larvae of lea-1 mutants. Figure S3. Mutations in lea-1 impact wild-type worms (N2) similarly to daf-2 mutants. Figure S4. LEA-1 does not significantly alter polyglutamine protein aggregation due to age. Figure S5. LEA-1 expression does not increase in response to short-term osmotic stress. Figure S6. Representative images of auxin-induced depletion of mNG::lea-1 in TIR1 expressing strains. Figure S7. Overexpression of lea-1a in body wall muscle does not rescue SDS sensitivity, desiccation tolerance, or osmotic stress resistance. Figure S8. Controls for heterologous expression of proteins in bacteria. Figure S9. Characterization of LEA-1 motif-expressing worms. Figure S10. Expression of LEA-1 motifs in body wall muscle does not improve SDS resistance, desiccation tolerance, or osmotic stress survival. Table S1. Genotyping primers for lea-1 genomic edits. Table S2. Lifespan analysis of lea-1 mutants. [file 12915_2021_1176_MOESM1_ESM.pdf]

Additional File 1

LEA motifs promote desiccation tolerance *in vivo*

Jonathan D. Hibshman[a] and Bob Goldstein[a,b]

[a]Biology Department, University of North Carolina at Chapel Hill, Chapel Hill, NC, USA

[b]Lineberger Comprehensive Cancer Center, University of North Carolina at Chapel Hill, Chapel Hill, NC, USA

*Corresponding author, Jonathan D. Hibshman

Biology Department

616 Fordham Hall

The University of North Carolina at Chapel Hill

Chapel Hill, NC 27599-3280

E-mail: hibsjo01@email.unc.edu

Figure S1

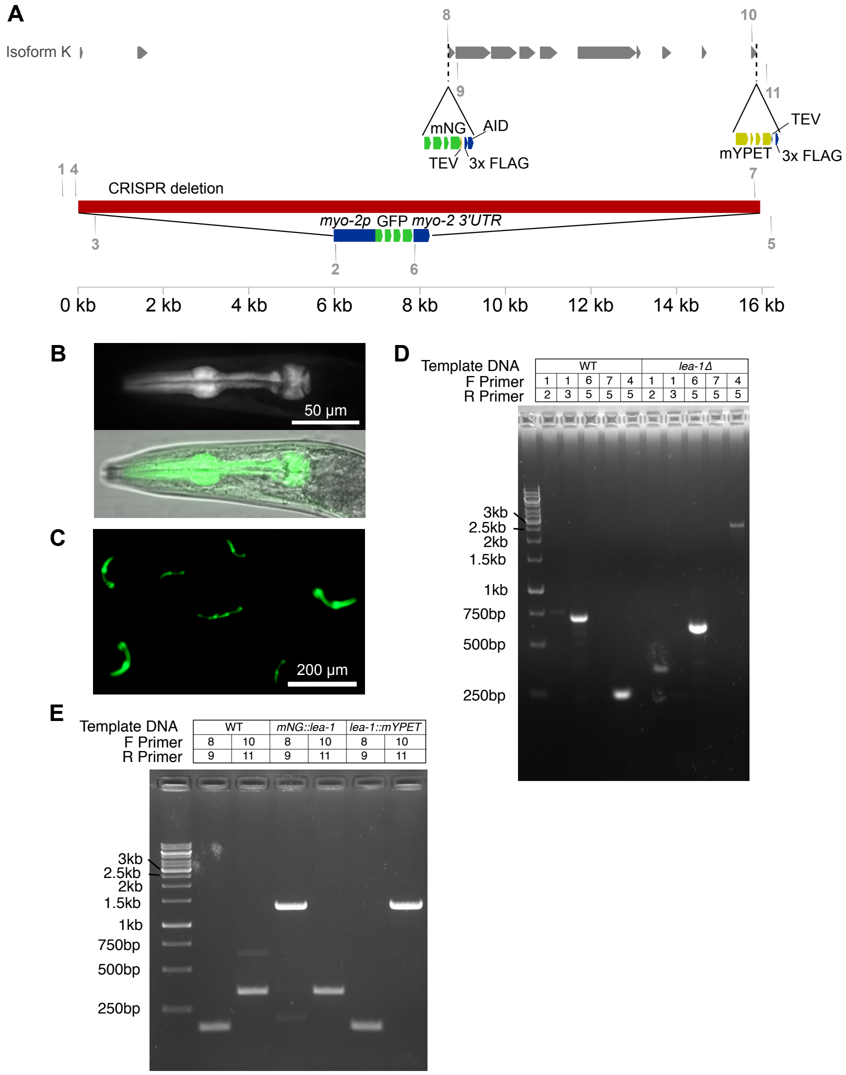

Additional File 1: Figure S1. Genotyping LEA-1 alleles. **A)** A simplified schematic depicting locations of primers to genotype the various genome edits. Primers are indicated with numbers. Sequences can be found in Table S1. **B)** A representative image depicts pharyngeal GFP expression in an *lea-1Δ* mutant in which 15.8 kb of genomic sequence was replaced with *myo-2p*::GFP::*myo-2 3'UTR* as a visual marker for the deletion. **C)** A representative image shows the plate level phenotype of pharyngeal GFP expression in *lea-1Δ* mutants. **D)** Primers as indicated by numbers in Figure 1 were used to amplify genomic DNA and confirm deletion of the endogenous *lea-1* locus and insertion of *myo-2p*::GFP::*myo-2 3'UTR* in the *lea-1Δ* mutant. **E)** The indicated primers were used to amplify across the loci in genomic DNA at which the mNG and mYPET tags were inserted. Genotyping confirms insertion of the mNG::3xFLAG::AID tag in a relatively N-terminal position of *lea-1* and insertion of the mYPET::3xFLAG tag at the C-terminus of the gene.

Figure S2

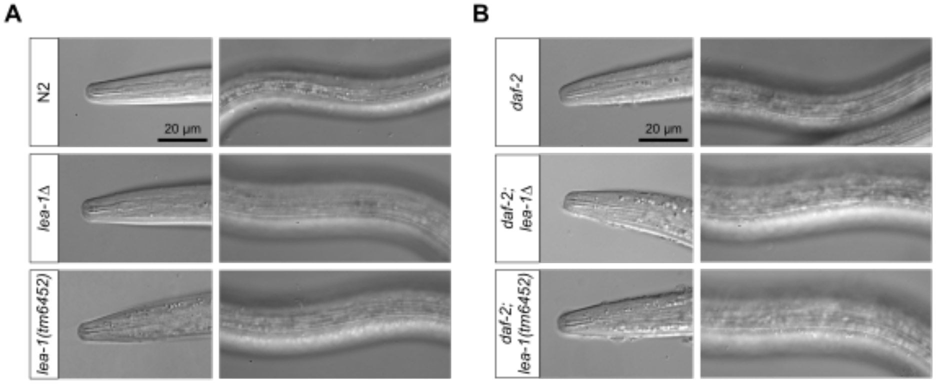

Additional File 1: Figure S2. Representative images of dauer-like larvae of *lea-1* mutants. **A)** The mouth and alae of N2, *lea-1Δ*, and *lea-1(tm6452)* worms are shown. **B)** The mouth and alae of *daf-2*, *daf-2;lea-1Δ*, and *daf-2;lea-1(tm6452)* worms are shown. Dauer-like larvae were formed at 25 ºC in both A and B.

Figure S3

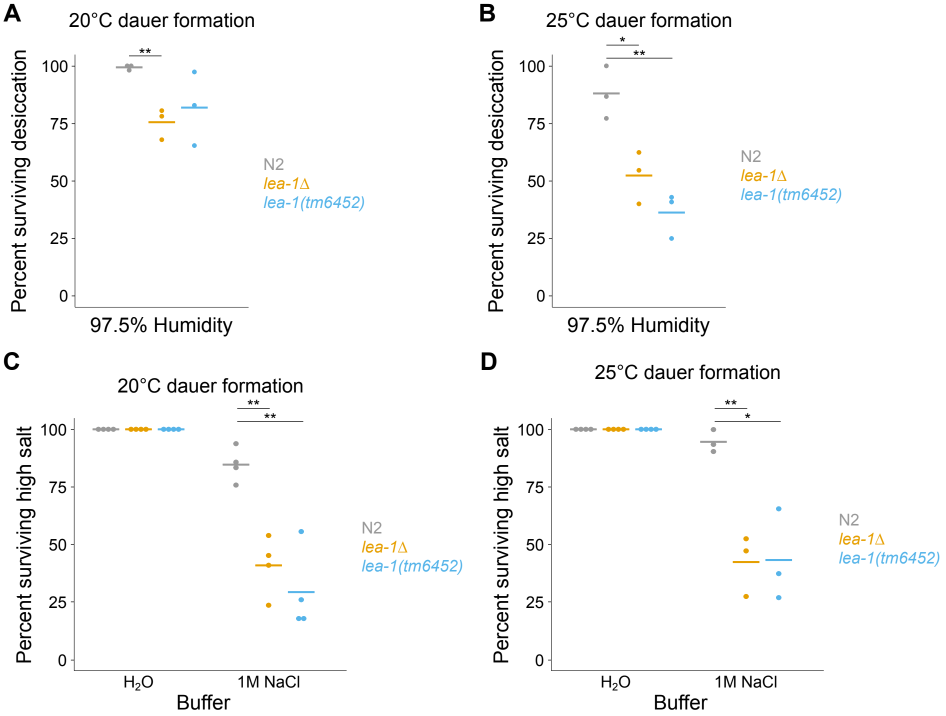

Additional File 1: Figure S3. Mutations in *lea-1* impact wild-type worms (N2) similarly to *daf-2* mutants. **A)** *lea-1Δ* dauer larvae formed at 20 ºC had reduced desiccation survival at 97.5% RH (p=0.003, unpaired T-test vs. N2, n=3). Desiccation survival of *lea-1(tm6452)* mutants was statistically indistinguishable from N2 (p=0.13, unpaired T-test, n=3). **B)** Both *lea-1Δ* and *lea-1(tm6452)* dauer-like larvae formed at 25 ºC have reduced desiccation survival at 97.5% RH (p=0.02, p=0.004 respectively, unpaired T-tests vs. N2, n=3). **C)** *lea-1Δ* and *lea-1(tm6452)* dauer larvae formed at 20 ºC have reduced survival in 1M NaCl for 2 hr (p=0.001, p=0.001, unpaired T-tests vs. N2, n=4). **D)** *lea-1Δ* and *lea-1(tm6452)* dauer-like larvae formed at 25 ºC have reduced survival in 1M NaCl for 2 hr (p=0.003, p=0.01 respectively, unpaired T-tests vs. N2, n=3).

Figure S4

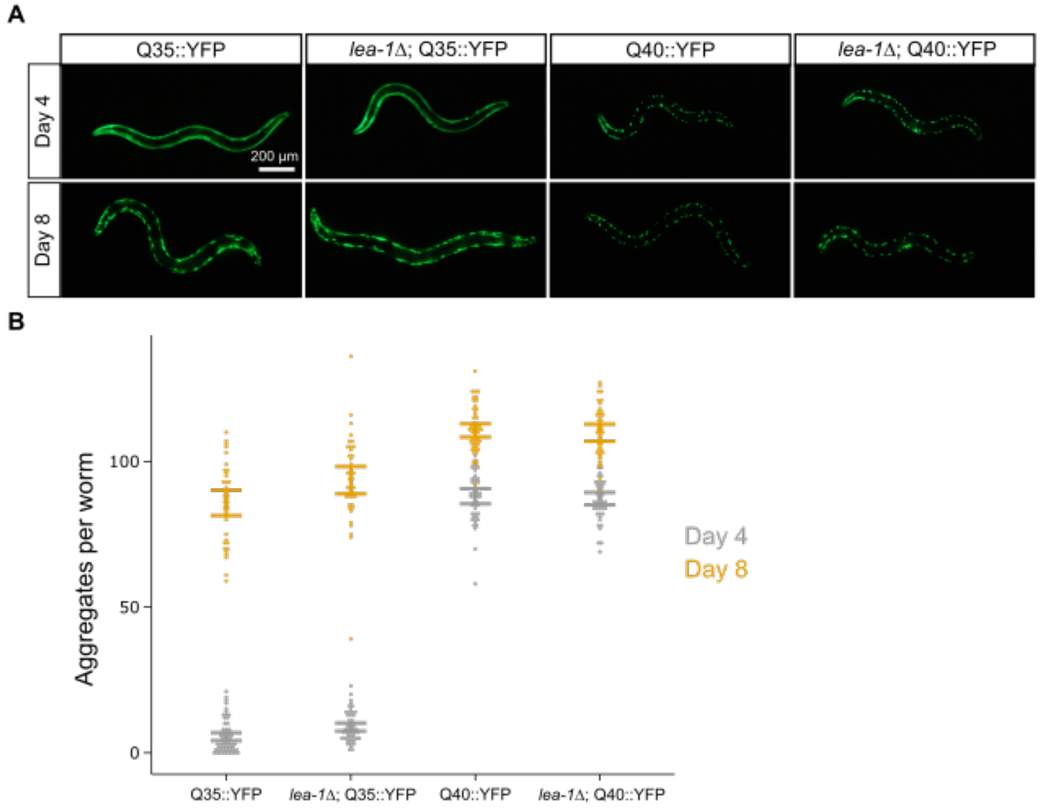

Additional File 1: Figure S4. LEA-1 does not significantly alter polyglutamine protein aggregation due to age. **A)** Representative images of 4 and 8 day old worms expressing polyglutamine::YFP constructs in body wall muscle. **B)** There are no significant differences in the number of polyglutamine aggregates between control (N2 background) and *lea-1Δ* animals for either Q35::YFP (day 4 p=0.23, day 8 p=0.42, n=3, unpaired T-test) or Q40::YFP (day 4 p=0.81, day 8 p=0.32, n=3, unpaired T-test).

Figure S5

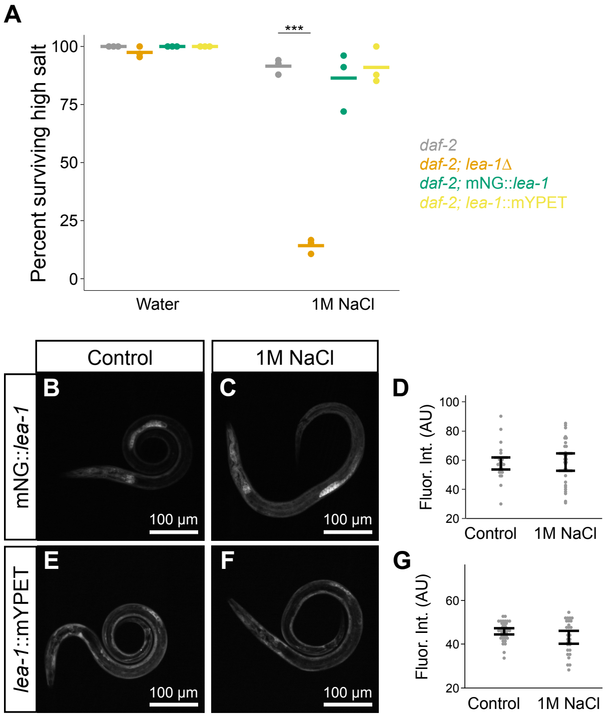

Additional File 1: Figure S5. LEA-1 expression does not increase in response to short-term osmotic stress. **A)** Fluorescent tags to not disrupt function of LEA-1 during osmotic stress. Survival is plotted for worms exposed to either water or 1M NaCl for 2 hr. Bars represent mean survival. Neither *daf-2;* mNG::*lea-1* nor *daf-2; lea-1*::mYPET worms were significantly different from control *daf-2* worms exposed to 1M NaCl. **B)** Representative image of a control *daf-2;* mNG::*lea-1* dauer worm. **D)** A *daf-2;* mNG::*lea-1* dauer larvae exposed to 1M NaCl for 2 hr. **E)** mNG fluorescence is not significantly altered in worms exposed to 1M NaCl relative to controls (p=0.86, n=3 replicates, unpaired T-test). **F)** A representative *daf-2* dauer worm expressing *lea-1*::mYPET. **G)** A *daf-2; lea-1*::mYPET dauer after 2 hr in 1M NaCl. **H)** Fluorescent intensity is not significantly different between controls worms expressing *lea-1*::mYPET (p=0.36, n=3 replicates, unpaired T-test). *** indicates p<0.001.

Figure S6

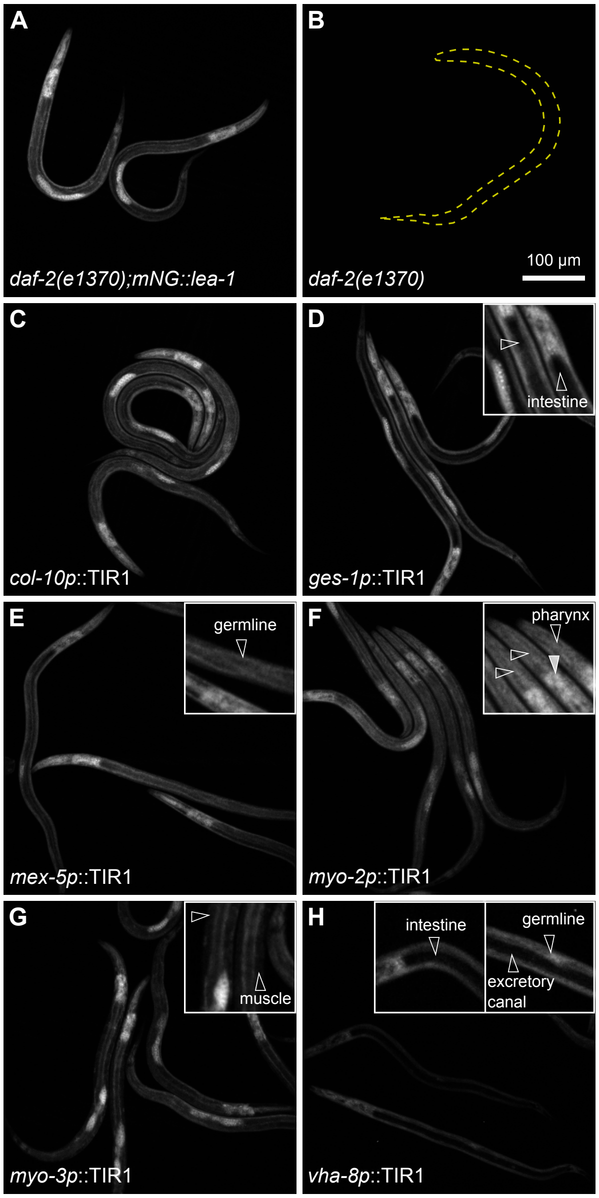

Additional File 1: Figure S6. Representative images of auxin-induced depletion of mNG::*lea-1* in TIR1 expressing strains. **A)** Baseline expression of mNG::LEA-1 in the absence of TIR1 expression. **B)** *daf-2* mutant animals have no detectable baseline fluorescence. Yellow dashed lines indicate the outline of a worm. **C)** TIR1 expressed from the *col-10* promoter should deplete LEA-1 in the hypodermis. It had a minimal effect on expression levels in worms. **D)** TIR1 driven by the *ges-1* promoter reduced LEA-1 in the intestine. **E)** TIR1 under the control of the *mex-5* promoter depleted LEA-1 in the germline. Some worms retained a significantly reduced amount of germline LEA-1. **F)** *myo-2p*::TIR1 worms have reduced LEA-1 in the pharynx. This construct did not totally deplete LEA-1. In particular, expression levels are still relatively high in the

posterior bulb of the pharynx (solid arrowhead). Depletion in the more anterior regions of the pharynx was more pronounced (open arrowheads). **G)** Expression of *myo-3p*::TIR1 significantly reduced LEA-1 levels in body wall muscle. **H)** TIR1 driven by the *vha-8* promoter is expressed in multiple tissues and depletes LEA-1 in the excretory cell (and canal), intestine, and germline. TIR-1 expression was also observed in hypodermis and some cells of the head. The 100 µm scale bar applies to all images. All images were taken with the same microscope settings and are shown on the same intensity scale. For the 2x magnified insets the brightness was adjusted to facilitate visualization. All worms were grown on plates containing 1mM auxin. Open arrowheads indicate sites of LEA-1 depletion in each strain.

Figure S7

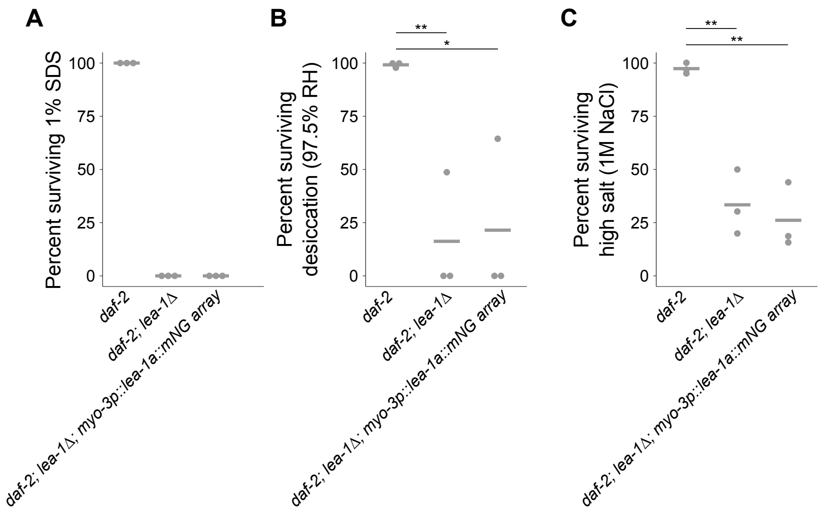

Additional File 1: Figure S7. Overexpression of *lea-1a* in body wall muscle does not rescue SDS sensitivity, desiccation tolerance, or osmotic stress resistance. **A)** Expression of mNG-tagged *lea-1a* in body wall muscle (*myo-3*p::*lea-1a*::mNG) does not increase 1% SDS survival of dauer-like larvae formed at 25 ºC. **B)** Body wall expression of mNG-tagged *lea-1a* does not improve desiccation survival at 97.5% RH. *daf-2;lea-1Δ* and *myo-3p*::*lea-1a*::mNG worms each have reduced desiccation survival at 97.5% RH relative to *daf-2* controls (p=0.007, p=0.02, respectively, n=3, unpaired T-test) **C)** Survival of osmotic stress in 1M NaCl for 2 hr is not improved by expression of *myo-3p::lea-1a::*mNG. Both *daf-2;lea-1Δ* and *myo-3p*::*lea-1a*::mNG worms are sensitive to 1M NaCl (p=0.002, p=0.001 respectively vs. *daf-2*, n=3, unpaired T-test). * indicates p<0.05, ** indicates p<0.01.

Figure S8

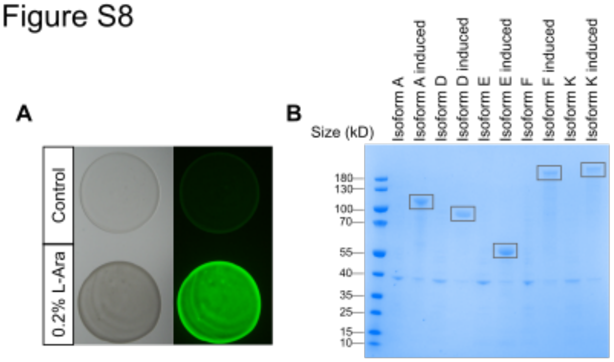

Additional File 1: Figure S8. Controls for heterologous expression of proteins in bacteria. **A)** BL21 *E.coli* carrying pDest17 driving expression of GFP are induced by growth in media with 0.2% L-arabinose. Samples of bacteria from liquid cultured were spotted onto LB agar plates for imaging. **B)** A Coomassie stained 4-12% Bis-Tris gel shows expression of *C. elegans* LEA-1 isoforms A, D, E, F, and K in BL21 *E. coli* when induced with 0.2% L-arabinose. 20 µg of total protein was loaded per lane.

Figure S9

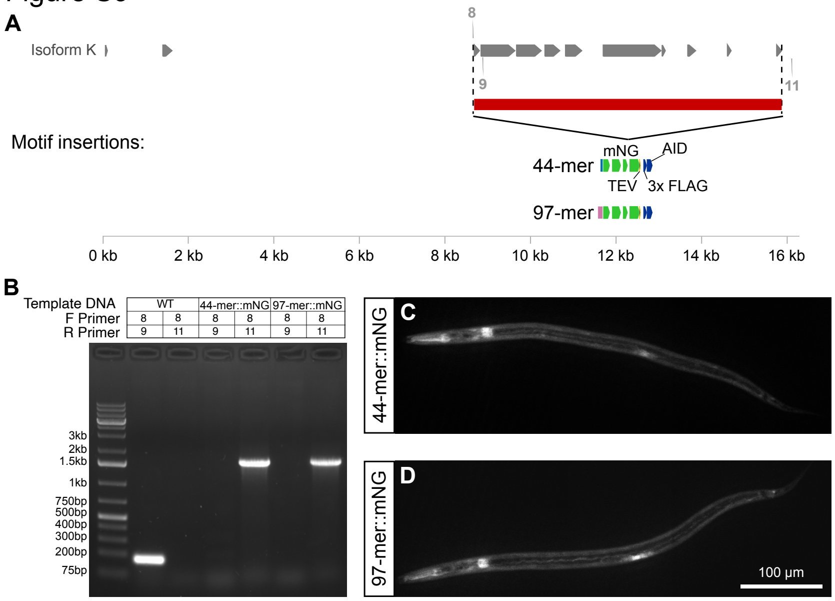

Additional File 1: Figure S9. Characterization of LEA-1 motif-expressing worms. **A)** A schematic depicts the regions of genomic deletion and insertions and the primers used for genotyping these edits. **B)** PCR genotyping confirms deletion of the majority of LEA-1 exons and insertion of sequence encoding 44-mer::mNG or 97-mer::mNG. **C)** A representative image depicts *in vivo* expression of the 44-mer::mNG fusion protein in a dauer worm. **D)** A representative image depicts *in vivo* expression of the 97-mer::mNG fusion protein in a dauer worm. Worms in C and D were in a *daf-2* background.

Figure S10

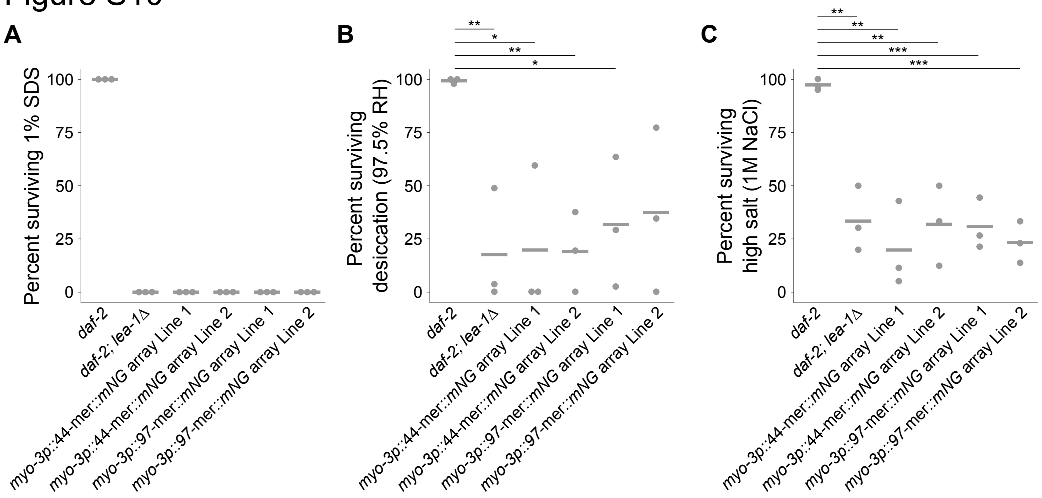

Additional File 1: Figure S10. Expression of LEA-1 motifs in body wall muscle does not improve SDS resistance, desiccation tolerance, or osmotic stress survival. **A)** Overexpression array lines carrying *myo-3p*::44-mer::mNG or *myo-3p*::97-mer::mNG transgenes remain sensitive to 1% SDS. **B)** *myo-3p*::44-mer::mNG and *myo-3p*::97-mer::mNG array lines do not rescue 97.5% RH desiccation survival of *daf-2;lea-1Δ* animals. Strains have reduced survival relative to *daf-2* controls (*daf-2;lea-1Δ* p=0.006, *myo-3p*::44-mer::mNG Line 1 p=0.02, *myo-3p*::44-mer::mNG Line 2 p=0.002, *myo-3p*::97-mer::mNG Line 1 p=0.02, *myo-3p*::97-mer::mNG Line 2 p=0.05, n=3, unpaired T-test). None of the array carrying lines is statistically distinguishable from *daf-2;lea-1Δ*. **C)** Muscle-specific expression of LEA-1 motifs does not improve survival of 2 hr osmotic stress in 1M NaCl. Survival of each array carrying line was not significantly different from *daf-2; lea-1Δ*. Rather, strains remain sensitive to 1M NaCl (*daf-2;lea-1Δ* p=0.002, *myo-3p*::44-mer::mNG Line 1 p=0.003, *myo-3p*::44-mer::mNG Line 2 p=0.004, *myo-3p*::97-mer::mNG Line 1 p=0.0007, *myo-3p*::97-mer::mNG Line 2 p=0.0002 vs. *daf-2*, n=3, unpaired T-test). * indicates p<0.05, ** indicates p<0.01, *** indicates p<0.001.

Additional File 1: Table S1. Genotyping primers for *lea-1* genomic edits.

Primer number	Sequence	Genotyping information
1	CGATGGTACCACAATGACCA	For use with primers #2 and #3.
2	TTTATGGACATTTAAAGCAAAGGA	Paired with #1 will give 417 bp band in *lea-1*Δ but no band in WT.
3	TCACTTGAGAGCCCGAACTT	Paired with #1 will give 742 bp band in WT but no band in *lea-1*Δ.
4	GATCACCATCTCCACCAACC	Paired with #5 will give 2,569 bp band in *lea-1*Δ but no band in WT.
5	GGGCAATCCAAAAAGATTGA	For use with primers #4, #6, and #7.
6	ACCCGTTTCTCTTCCCCTAC	Paired with #5 will give 620 bp band in *lea-1*Δ but no band in WT.
7	GGACACTCTTCGCTCGACTC	Paired with #5 will give 256 bp band in WT but no band in *lea-1*Δ.
8	CAACAAAATGAGCTTTATGGATAAAG	For use with primer #9.
9	ATCTTTTCACCGACGGTGTTG	Paired with #8 will give 170 bp band in WT and 1,364 bp band in mNG::*lea-1* but no band in 44-mer::mNG or 97-mer::mNG.
10	ACTTGAAAACTGGAACTCTTCCATC	For use with primer #11.
11	CTACAACAAAGAATTAACAG	Paired with #10 will give 347 bp band in WT and 1,412 bp band in *lea-1*::mYPET. When paired with #8 will give 1,612 bp band in 44-mer::mNG and 1,771 bp band in 97-mer::mNG.

Additional File 1: Table S2. Lifespan analysis of *lea-1* mutants.

Genotype	n	Mean Survival (days)	SE	95% CI	Maximum Survival (days)
N2	151	13.22	0.38	12.47 ~ 13.96	27
lea-1(tm6452)	150	11.94	0.3	11.35 ~ 12.53	21
lea-1Δ	150	13.75	0.42	12.91 ~ 14.58	27
daf-2	141	34.36	1.47	31.48 ~ 37.23	75
daf-2;lea-1(tm6452)	144	26.29	1.77	22.83 ~ 29.76	73
daf-2;lea-1Δ	150	36.89	1.56	33.84 ~ 39.95	73
